# Are patients ready for discharge from the hospital after fast-track total knee arthroplasty?-A qualitative study

**DOI:** 10.1371/journal.pone.0303935

**Published:** 2024-05-29

**Authors:** Simeng You, Na Li, Manjie Guo, Hong Ji

**Affiliations:** 1 The affiliated hospital of Jiaxing University, The First Hospital of Jiaxing, Jiaxing, Zhejiang, China; 2 Nursing department, The First Affiliated Hospital of Shandong First Medical University, Jinan, Shandong, China; 3 Beijing Hospital of Traditional Chinese Medicine, Capital Medical University, Beijing, China; 4 School of Nursing and Rehabilitation, Shandong University, Jinan, Shandong, China; Stanford University School of Medicine, UNITED STATES

## Abstract

**Background:**

The fast-track based on evidence-based medicine, has dramatically reduced the length of stay for patients undergoing total knee arthroplasty (TKA). Therefore, patients must assume the responsibility for self-functional exercise and care as early as possible. Also, higher standards and expectations of care delivery have been set. Studies into patients’ experiences when faced with a discharge decision under a fast-track program are lacking.

**Objectives:**

(1) Increase the knowledge about patients’ experiences of discharged from hospital via a fast-track process after TKA. (2) Explore what gaps exist in the current discharge preparation care service for TKA under fast-track and what can be improved.

**Methods:**

A qualitative research design was chosen to conduct semi-structured face-to-face interviews with 21 patients from one Chinese hospital who successfully underwent TKA and received discharge orders. Interview data were meticulously analyzed, summarized and thematically distilled using Interpretative Phenomenological Analysis (IPA).

**Results:**

Three themes emerged from the structural analyses: a) Preparing for discharge despite concerns about symptoms-a sense of joy at discharge despite feelings of helplessness, stigmatisation, anxiety about prosthetic function. b) Managing the rehabilitation difficulties-vigilance is needed for medication management, environmental changes, and intimate relationships. c) Creating conditions for safe transition-compassionate bedside manner, listening to patients, and providing a humanized continuing care and referral services are important for safe transitions.

**Conclusion:**

Findings suggest that patients undergoing fast-track TKA report good discharge preparation experiences. However, closer analysis reveals difficulties with this process and important directions in which discharge readiness care services can strive.

## Introduction

TKA is considered the most effective treatment for severe end-stage knee osteoarthritis, addressing severe pain, impaired joint function, significant deformities, and instability resulting from joint diseases [1, 2]. Using available 2000–2014 NIS figures, the U.S. projects that primary TKA is projected to grow 85%, to 1.26 million procedures, by 2030 [3, 4]. In China, there has also seen a rapid rise in TKA surgeries, escalating from 50,000 cases annually a decade ago to nearly 400,000 cases [5].

“Fast-track” [6] refers to a well-established, safe perioperative care strategy involving multidisciplinary collaboration. Since its introduction in the late 1990s, it has gained widespread acceptance for its ability to achieve several key benefits, including shorter hospital stays [7, 8], reduced mortality [9], and fewer postoperative complications [10]. It is now recognized as a standard of care in orthopedics [11]. Consequently, with the increasing number of TKAs in China, the concept of fast track surgery (FTS) has been widely adopted in this field.

While reaping the clinical benefits of this approach, it’s essential to remain vigilant about the challenges associated with centralized clinical pathways. Firstly, shorter hospital stays for TKA patients do not equate to reduced perioperative care but instead require significant organizational and administrative efforts by healthcare staff involved in patient discharge [12]. Additionally, patients are also expected to assume responsibility for engaging in functional exercises and self-care as early as possible, with their readiness for discharge being critical to ensure the continuity of subsequent rehabilitation. Previous studies consistently highlight that patients frequently don’t feel adequately prepared for discharge [13], with limited attention to patients’ subjective experiences [14]. Rushed discharges have been linked to elevated readmission rates [15], increased postoperative complications, and unexpected healthcare resource utilization [16]. Weiss [15] and Coffey [17] have advocated that incorporating patients’ own perspectives in evaluating discharge readiness reduces unplanned readmission and mortality rates, as well as optimizing the discharge planning process while improving patient satisfaction. Recognizing that information is closely tied to trust, it is essential to prioritize listening to and understanding the signals conveyed by patients.

Current studies have predominantly focused on patients’ experiences related to pain and rehabilitation after short-term discharge, there appears to be limited understanding patients’ experience and needs at the time of receive discharge instructions. It is reasonable to assume that the stress and concerns expressed by patients when facing the decision to be discharged will be more reflective of the current care challenges. This study aims to describe patients’ experiences of discharged from hospital following TKA under FTS in China. Additionally, this study attempts to explore what gaps exist in current discharge preparation care service and what could be improved.

## Methods

### Design

This study is a qualitative research methodology that employs Interpretative Phenomenological Analysis (IPA), which aims to gain a deeper understanding of individual experiences and subjective perceptions. This method is typically used to explore how individuals interpret and ascribe meaning to events, emotions, and phenomena they encounter [18]. Furthermore, it has gained prominence and widespread use over the past decade to fifteen years [19]. The focus of this study is to delve into the subjective construct of the discharge experience as perceived by each participant, which encompasses recollections, impressions, sensations, and fragmentary statements of others. This aligns well with the strengths and capabilities of IPA.

To ensure rigorous and transparent reporting of the research process, the study adhered to the Consolidated Criteria for Reporting Qualitative Research (COREQ) checklist [20] (S1 Checklist).

### Participants

We purposively recruited patients who underwent fast-track TKA between May 2023 and July 2023 in the orthopaedic wards of a tertiary A-level hospital located in Jinan, Shandong Province, China. Patients with ASA classification of class I, II and stable III were enrolled in the fast-track program (S1 File). The hospital is a provincial general hospital open to the public with a specialist orthopaedic inpatient unit and a systematic, standardized perioperative care process specializing in the treatment of joint replacements, joint revisions and wound infections. Patients of different ages, educational levels, and living situations were selected to ensure a diverse sample. The inclusion criteria were as follows: a) receive primary unilateral TKA for advanced KOA; b) under the fast-track program; c) receipt of a discharge order; d) demonstrate effective communication skills; and e) willingness to join the study and consent in writing.

The sample size was determined by considering the participants’ ability to provide relevant information, ensuring that each participant’s data met the study’s requirements [21]. Furthermore, Morse et al. [22] demonstrated that when using a semi-structured interview approach, each participant tends to provide a more constrained and limited account of their experiences compared to an open-ended interview. As a result, in this study, a larger number of interviews was deemed necessary to achieve data saturation. Data saturation is reached when no new themes or crucial information emerge from the interviews.

### Procedure

In this study, all interviews were conducted by a female author (SMY), a third year student for a Master’s degree in Nursing at University. Prior work experience of six months in an orthopaedic internship. Received extensive theoretical assessment on qualitative research from the School of Nursing and systematic training on interviewing skills from the First Affiliated Hospital of Shandong First Medical University. None of the participants were known to the interviewer.

Patients were warmly invited to participate in interviews when the researcher (NL) received and verbally explained the discharge instructions (S2 File). Patients were provided with comprehensive information about the study’s purpose, significance, and process, and were invited to participate if they expressed interest. Interviews were conducted within 24 hours of receiving the physician’s discharge order. To establish a connection with the participants and encourage them to openly express their feelings and thoughts, the interview began with general questions. Throughout the process, the interviewer (SMY) actively suspended preconceived thoughts or experiences, adhering to the principle of attentive listening. Face-to-face interviews were conducted in a relatively comfortable and quiet departmental conference room, typically involving one participant at a time and lasting approximately 30–45 minutes. To ensure construct validity in the qualitative interviews, a pilot study was conducted on three patients. The final interview guide was co-developed by all the authors based on the research questions, and careful consideration was given to all issues in this area, which included the patient’s surgical situation and outcome assessment, experience and assessment of discharge, and consideration of the patient’s support level and dilemmas in terms of caregivers, family members, and social support, etc. To minimize the likelihood of missing any potentially relevant factors (e.g., pain management, surgical wound treatment, respiratory therapy, and proper medication were listed as medical factors affecting the later stages of discharge readiness) were pre-included to elicit more thought and response during the pilot study. The "yes or no" questions were further designed with open-ended questions (e.g., Do you feel ready for discharge? If not, what do you think needs to be done better?) The complete interview guide is available in S3 File.

### Data collection and processing

Data collection and processing were carried out by two researchers (SMY and NL). Data were gathered using various tools, such as mobile phone audio, pen and paper notes during the interview process. Additionally, meticulous attention was paid to observing and documenting the patients’ voice tones, expressions and body movements.

Within 24 hours of conducting the interviews, two researchers collaborated in transcribing, coding and analyzing the recorded content. The data were transferred to NVivo11 software for this purpose. The primary author (SMY) carefully read through each text multiple times to develop a comprehensive understanding of the content. Subsequently, meaningful statements within the text were highlighted to capture the essential elements. These significant elements were then condensed into clear and concise units of meaning while considering their contextual connections. Units with similar meanings and recurring instances formed sub-themes. Finally, the underlying meanings within the sub-themes were contemplated, leading to the identification of phenomena that could serve as relevant headings, which were then unified as themes [23, 24]. The second author (NL) was engaged in the process from its inception, including the design phase of the research questions and interview guide. Throughout this process, the main responsibility was to review notes and provide comments on the main findings, and if necessary, follow-up interviews as needed to verify the results. Consistency in the results was achieved through the collaboration of both researchers and the comprehensive description of the data, thereby confirming the transfer ability of the findings.

### Ethical considerations

The Ethics Committee of the School of Nursing and Rehabilitation at Shandong University approved this study (No. 2023-R-021). Rigorous adherence to ethical principles was maintained throughout the study. Informed consent was diligently obtained from all participants, with researchers providing a clear and unconditional explanation of the study’s objectives. A solemn commitment to confidentiality was made, and participants were informed of their right to withdraw from the study at any time without any consequences. To protect the privacy of the patients, their names were anonymized with strict measures in place.

## Results

### Characteristics of participants

The final analysis included all 21 participants, with no dropouts. Their ages ranged from 53 to 80 years, and the majority of patients were female, living only with their spouses, and were usually interviewed on the postoperative day 3. Table 1 presents the self-reported demographics and characteristics of the participants. Data saturation was achieved after conducting 21 interviews, with no new data emerging thereafter. No repeat interviews were conducted.

**Table 1:** Demographic and characteristics of the participants (N = 21).

Participants		n = 21
Sex	Male	8
	Female	13
Age, years	Median	68
	Range	53–80
Educational levels	Elementary school and below	11
	Junior high school	6
	High school (including junior college)	2
	University (including college) and above	2
Marital status	Married	17
	Widowed	4
Living arrangements	Only with spouse	12
	With family members	5
	Alone	2
	With live-in care worker	2
Residence area	City	12
	Rural	9
Hospital length of stay, days	Median	3
	Range	2–5
Number of chronic diseases	0	6
	1~2	9
	3	6

### Overview of themes

The interview process naturally provided a comprehensive understanding of patients’ experiences of preparing for discharge, three themes emerged:1) Preparing for discharge despite concerns about symptoms, 2) Managing the rehabilitation difficulties, and 3) Creating conditions for safe transition. The S1 Table illustrates how these themes emerged.

#### Preparing for discharge despite concerns about symptoms

*Satisfaction with improved functioning and a sense of reborn joy*. In the interview reports, patients expressed satisfaction with the improvement in knee function.

“I feel like I’m healing day by day…(The third day after the surgery)” (ZC, Male, 68 years old, Post-op day 4).

“my surgeon told me that my test results were good, and rehab exercises were on track…” (GWX, Male, 57 years old, Post-op day 3)

Patients tend to compare themselves to fellow patients or sense the beginnings of an impending discharge from conversations with nurses or rehabilitation physicians when making their self-assessment.

“The guy next to me had his surgery a day before me, and he got sent home yesterday…I guess it’s probably my turn to go home.” (ZYJ, Female, 63 years, live only with spouse, Post-op day 3)

“The physician said that I can bend (90°) and straighten (180°) my knee now, I would be out of the hospital soon.” (LW, Male, 69 years old, Post-op day 4)

As the topic of discharge came up, they felt a sense of reborn joy. Expressions such as “going home comes first” and “looking forward to being discharged” were frequently used.

“The surgery went well… Getting home is what I’m really looking forward to.” (ZTA, Male, 66 years, live only with spouse, Post-op day 3)

“I’m all set to head home now, going home comes first.” (ZYX, Female, 54 years, live only with spouse, Post-op day 2)

*Feeling of helplessness*. The influx of information or heightened sensitivity to physical symptoms often generated a sense of vigilance and uncertainty among participants who were overwhelmed.

“It happened so quickly… If only they had let me stay one more day, I think I could have absorbed all this info…” (SXQ, Female, 69 years, live alone, Post-op day 3)

“I’m uncertain about… cause the skin on this leg is numb and doesn’t quite feel like mine, but maybe that’s just part of the deal.” (WTY, Female, 61 years, live with family members, Post-op day 2)

Participants also highlighted their feelings of helplessness in the face of future self-care.

“I’m not so sure I would have done subsequent rehab movements on my own. A sense of being left to its own devices.” (DJ, Female, 71 years old, Post-op day 3)

*Stigmatisation*. Participants also tend to associate these physical body image changes with daily socialisation after discharge, which can be potential stigmatising for them.

“They might think I’m in the same boat as someone with a disability.” (ZYX, Female, 54 years, live only with spouse, Post-op day 2)

In turn, they argued that the majority of people on the ward had undergone joint surgery and that there were similarities between them, therefore they would not be viewed differently.

“In the hospital, we’re all in the same boat, and there’s no judgment like that (discriminated against)”. (CBC, Female, 78 years old, Post-op day 3)

*Anxiety about prosthetic function*. As most of the participants lacked a medically relevant social background and were undergoing joint replacement surgery for the first time, they voiced anxiety regarding the functionality and durability of their prostheses.

“Can I really go for a deep squat? Could it possibly mess up this fake knee joint? I’m not entirely convinced this artificial joint is as great as the doc claims it to be.” (DJ, Female, 71 years, live only with spouse, Post-op day 3)

“I’m kinda scared that if I push it too hard, I might end up needing another replacement…” (ZYJ, Female, 63 years old, Post-op day 3)

#### Managing the rehabilitation difficulties

*Highly motivated and currently on the road to recovery*. Postoperative rehabilitation requires continuous and incrementally challenging training. Patients need to possess exceptional motivation and discipline as essential qualities. Interviews revealed that patients were spurred to enact constructive changes by their experiences of traumatic growth. They expressed a keen willingness to adhere to self-management recommendations and exhibited a strong drive to persevere in their rehabilitation regimen, thus embarking on a path to recovery.

“I’m feeling great and totally ready to keep up with the exercises, even if it means dealing with some intense pain while at home this time.” (YMY, Female, 65 years, live only with spouse, Post-op day 2)

“This surgery is a big deal in my life, I’m absolutely committed to my post-surgery rehab…” (GXP, Female, 68 years, live only with spouse, Post-op day 3)

“Post-op rehab is a big deal, and I can’t afford to lose in this battle.”(YM, Male, 74 years old, Post-op day 3)

*Medication management*. Nevertheless, various unsettling and disruptive factors can pose significant barriers. These include a biased comprehension of medication dosages, such as excessive caution or relying on painkillers.

“I barely noticed the minor pains, and as long as it’s not particularly painful, I’ll opt out of pain medication because painkillers are a chemical.” (ZWQ, Female,73 years, live alone, Post-op day 4)

“I’ll admit, I was feeling scared. Requested my doctor to give me an extra dose of painkillers…”(LYQ, Male, 56 years old, Post-op day 2)

Actually, most patients do not realise that the pain can be intense, especially at night.

“Well rested is important, (The first night after surgery) I didn’t really get into a deep sleep and the pain got worse, it’s quite a struggle.” (XZZ, Female, 68 years, live with other family members, Post-op day 3)

*Environmental changes*. The hospital ward environment can be advantageous for patients, and the insufficient adaptation of their homes for post-surgery recovery frequently leads to delayed recuperation following hospital discharge. Ensuring a safe environment for conducting activities was a significant consideration for the participants.

“I mean… at home there isn’t a strict no-smoking policy like there is in hospitals, where everything around you has handles and is sterilised regularly.” (DJ, Female, 71 years, live only with spouse, Post-op day 3)

“That walker won’t fit in our bathroom at home, and I’m worried I might take a tumble without it.” (JH, Female, 53 years, live only with spouse, Post-op day 2)

The change in surroundings also signifies a shift in dietary patterns and lifestyle habits, and participants also raised concerns related to home-cooked meals.

“My doc and nurse told me to stick to a high-protein and light diet. But you know, I’m a fan of spicy food.” (XLE, Male, 62 years, live only with spouse, Post-op day 3)

*Strained social and intimate relationships*. The majority of participants reported a significant reduction in socializing with friends and family relatives due to limitations in mobility and feelings of frustration.

“I won’t be attending for too many gatherings because I hardly go out on my own anymore.” (YS, Male, 67 years, live only with spouse, Post-op day 3)

An “ideal spousal model of recovery” resulted in spouses often taking on the important role of providing informal care free of charge, and participants mentioned that they suspended some physical intimacy to recover from the illness, but that did have an impact on the intimacy of the couple.

“My husband… tried not to share the same bed with me… he was worried about accidentally hurting my wounds during the night… Sometimes, it does get a bit lonely.” (XGB, Female, 69 years, live only with spouse, Post-op day 3)

Moreover, participants exhibited greater sensitivity to the impact of their illness and were often conscious of the concessions made by their family members to provide care, including time off work, financial support, and physical assistance, which engendered a sense of indebtedness.

“My son burned through all his saved-up vacation days… he’s given up his precious time and spent a lot of money.” (LW, Male, 69 years, live with other family members, Post-op day 4)

“my daughter was busy with work…but she put a lot of effort into this.” (LYQ, Male, 56 years old, Post-op day 2)

#### Creating conditions for safe transition

*The caring bedside manner of the nursing staff*. Participants generally perceived the management information provided by individuals with expert knowledge, such as attending physicians, rehabilitation specialists, and nurses, as reliable. Participants’ perceptions of their illnesses were largely derived from explanations given by this group, and participants indicated that it was the attentive bedside manner of caregivers that created predictability and confidence in the patient’s rapid discharge from the hospital.

“They actually came right to my bedside, kept repeating instructions in case I forgot, treating me like one of their own family…” (GYH, Female, 80 years, live with live-in care worker, Post-op day 5)

“The nurses taught me how to move, demonstrated with their own bodies at my bedside and constantly shared success stories of other patients… that really boosted my confidence.” (GWX, Male, 57 years, live only with spouse, Post-op day 3)

#### Listening to patients

In this study, participants emphasized the significance of the care team listening to their input and desires, as evidenced by communicating the timing of medication changes and choosing more appropriate means of communicating health information.

“I didn’t want to start rehab before the med change because I knew it would make the pain worse… The nurse understood my concern and shifted the schedule.” (XGB, Female, 69 years, live only with spouse, Post-op day 3)

“The nurses handed us a bunch of booklets, but I’ve got presbyopia. So, they hooked me up with a bunch of rehab videos…” (ZTA, Male, 66 years, live only with spouse, Post-op day 3)

Participants reported that when the care team learnt about their needs, they were given special consideration and felt valued.

“The benches in the ward were too low for me, probably because of my height (187 cm)…I mentioned this to the nurse, and she somehow managed to find me a taller chair.” (XLE, Male, 62 years old, Post-op day 3)

*A humanised continuing care and referral services*. Backed by a humanised continuing care and referral services. Patients attempt to establish communication channels with liaison officers to address their concerns after discharge.

“I was wondering if I could have their contact info because they know my health situation better than anyone else.” (SXQ, Female, 69 years, live alone, Post-op day 3)

In addition, participants indicated that they were informed of the rehabilitation progress to be followed up by outpatient review and telephone follow-ups, which will contribute to achieve a seamless transition.

“Luckily, the doctor created a WeChat group for us in advance. we can see the time when the attending doctor sits, and go to the outpatient clinic for review when the time for review arrives.” (LYQ, male, 56 years, live only with spouse, Post-op day 2)

## Discussion

This study presents the discharge readiness experience of patients undergoing fast-track TKA. It adds valuable knowledge about the understanding of how patients treated with TKA under fast track setting when facing discharge, in addition to revealing the difficulties patients face, it also adds some positive responses about clinical care practices.

The majority of participants reported favorable postoperative recovery experiences and expressed a readiness to be discharged from the hospital. This is similar to the findings of previous studies [25, 26] where the majority of patients reported that it was good to go home and take responsibility for their recovery. There are two plausible explanations for these positive results. First, it is possible that the perioperative multidimensional education program and the multidisciplinary collaborative management approach implemented within the framework of the fast-track concept facilitated patients in tapping into their potential to manage their conditions and acquire skills during the recovery phase [25]. Second, besides the cost of restorative surgery, bed occupancy, nursing services, and medical treatment during postoperative hospitalization place a similar financial burden on the patient. Most cost-effectiveness studies [27, 28] have used data on length of stay to calculate costs. The length of a patient’s postoperative hospital stay is considered a good indicator of healthcare costs, and the prospect of early discharge as a cost-effective rehabilitation option may have motivated them to prepare for discharge. Significantly, behind their positive response to the prospect of discharge, it became evident that various psychological issues were at play, including feeling of helplessness due to uncertainty about the medical condition, fear of social stigma, and heightened concerns about knee prosthesis. The same paradox was found in the study by Woolhead [29], where individuals reported their outcome from TKR as good, but further discussion revealed concern and discomfort with continuing pain and mobility difficulties. Such findings highlight a number of important issues related to the evaluation of discharge readiness. A mixed study [30] conducted in the Malaysian exploring the needs of orthopedic patients at discharge, found that there were emotional, physical and spiritual preparation needs. What’s more, in a study of nurses’ and patients’ perceptions of the discharge readiness, respectively, it was found that nurses rated discharge readiness higher than patients did [31]. These revelations prompted a reconsideration of the healthcare model in place, raising questions about whether a genuinely “patient-centered” [32] approach was being followed and whether healthcare professionals might have overestimated patients’ readiness for discharge while potentially overlooking their underlying mental health issues. In light of these findings, we offer several recommendations. Firstly, we recommend that healthcare professionals prioritize open dialogue with patients and remain attentive to the emotional fluctuations within the patients’ inner world. Secondly, it is essential to provide more comprehensive and detailed guidance to enhance patients’ confidence in coping with feelings of helplessness, stigma, and anxiety about prosthetic function. Additionally, we suggest that, during the postoperative hospitalization period, the flow of information should be managed thoughtfully to prevent overwhelming patients with excessive information.

Our findings also imply that patients’ readiness for discharge from hospital is not solely orientated by the caring behaviors of individual healthcare professionals. It is also influenced by the patients’ subjective will, pain management, changes in their surroundings, and support from family and friends. In this study, we were pleased to hear that patients were strongly motivated to undertake rehabilitation activities, such as “willingness to persevere without compromise”. However, this strong intrinsic motivation may have been somewhat countered by patients’ resistance to use analgesics and the challenges posed by the external environment and social relationships. For example, some patients didn’t take pain management seriously and resisted using analgesics, despite repeated messages conveyed to them that pain should be nipped in the bud. Additionally, a study similarly revealed that with regard to medication management, some patients held varying interpretations of medication adherence or considered alternative therapies, although they expressed intentions to follow prescribed medication schedules [33]. In this regard, it has been pointed out that optimization and reduction of pain is prerequisite for further development of TKA with rapid rehabilitation [34]. These findings caution that discharge planning teams should comprehensively consider the patient’s social context, assess their health literacy regarding pain management, efficiently coordinate analgesic prescriptions and medication regimens, and provide tailored education on medication use [35]. Furthermore, it’s crucial to consider the patient’s surrounding environment and family support. Our analysis revealed a recurring pattern of inadequate support, responding to gradual tension in family relationships and challenges in adapting to an uncertain living environment, aligning with findings by McKeown [36]. When examining the underlying causes, it became apparent that elderly participants, characterized by vulnerability and reduced mobility, often assumed a passive role in healthcare activities, faced difficulties in comprehending medical information, and frequently sought reassurance and assistance from external sources. These findings underscore the significance of the family’s role as a valuable resource in ensuring post-discharge care [30, 37]. Therefore, we recommend that healthcare professionals actively involve the family in developing home rehabilitation plans and conveying self-management instructions [38]. Additionally, it’s crucial to assess whether patients have access to external support, such as environmental modifications that promote safe activities [39].

Through a review and analysis, we’ve identified a “know-do gap” whereby our resident clinicians and nurses sometimes struggle to execute tasks despite knowing what should be done [40]. This “know-do gap” is also observed in our cultural context, where the final decision to discharge may be sudden, and standardized discharge care processes are challenging to implement fully due to understaffing in our hospital beds [41]. However, from the interviews conducted for this study, it appears that the current nursing teams effectively utilize the hospitalization period. They exhibit valuable qualities such as bedside manner, attentive listening to patients’ voice, and a humanized continuing care and referral services, which help bridge this gap. Increasingly, academia is recognizing the importance of valuing patients’ experiences and preferences in delivering high-quality care [42–44]. Attentive bedside manner and listening to patients allows healthcare providers to respond promptly to their urgent needs [45]. This leads us to the idea that empathy and the ability to think differently in healthcare professionals can be learned and impacted as a teachable communication skill rather than a personality trait. Finally, in order to better help patients achieve a smooth transition, we have to consider the patients’ needs for continuity of care and referral services. Encourage medical institutions to give full play to their advantages in professional technology and talents, provide patients with humane and diversified nursing services, gradually improve the content and mode of services, including but not limited to WeChat-based instant information exchange platform to ensure the continuity of care services.

### Strengths and limitations of the study

This study has several limitations that should be acknowledged. Firstly, the participants were recruited exclusively from one hospital, and the results are limited to the Chinese health care system. This limitation might raise concerns about the generalizability and broader significance of the findings. However, by selecting patients of different ages, educational levels, and living conditions, as well as pre-surveying prior to the commencement of the formal interviews to try to safeguard the transferability of the findings. Secondly, while this study aimed to identify deficiencies in the discharge services provided by the care team and develop relevant recommendations, it did not include interviews with care team members, particularly care managers. Relying solely on the patients’ perspective might restrict the applicability of the recommendations, but the results of this study are still instructive. In future research, it would be valuable to combine experiences of patients with those of healthcare professionals to gain a comprehensive understanding of priorities for service improvement. Lastly, despite interviewers clearly explaining to participants that they were not responsible for physical health assessments within the unit, participants may have still reported what they thought the researchers wanted to hear. This potential bias should be considered when interpreting the study’s findings.

In addition, this study possesses notable strengths. Firstly, the interview questions were intentionally designed to be open-ended, allowing participants the freedom to express their discharge preparation experiences in greater depth and detail. Secondly, the interviews were conducted promptly, within 24 hours of the doctor’s discharge instructions. This approach helped mitigate the potential recall bias that could arise over time. Furthermore, it is reasonable to assume that patients’ responses, obtained when they faced the immediate prospect of discharge, provided a more real-time and emotionally responsive perspective on the issues related to discharge preparation care delivery.

## Conclusion

Findings suggest that patients undergoing fast-track TKA report good discharge preparation experiences. However, closer analysis reveals difficulties with this process, highlighted by psychological activities such as feelings of helplessness, stigma, and anxiety associated with uncomfortable symptoms. Pain control, attention to environmental modifications and strengthening of social and family support are important to improve postoperative management. Finally, discharge preparation nursing services can continue to work on a cordial bedside manner, listening to the patient, and humanizing the continuum of care or referral services.

## Supporting information

S1 Checklist Consolidated criteria for reporting qualitative research (COREQ) checklist. (PDF)

S1 File Details of the fast-track clinical components. (DOCX)

S2 File Discharge information for patients. (DOCX)

S3 File Patient interview schedule. (DOCX)

S1 Table The structural analysis and how the themes and subthemes emerged. (DOCX)

## References

[1] Hamelynck KJ. The total knee prosthesis: indications and complications. Ned Tijdschr Geneeskd. 1998 Sept 12;142(37):2030–4. 9856207

[2] Rathjen KW. Surgical treatment. Total knee arthroplasty. Am J Knee Surg. 1998 Jun 01;11(1):58–63. 9533057

[3] Singh JA, Yu S, Chen L, Cleveland JD. Rates of total joint replacement in the United States: future projections to 2020–2040 using the National Inpatient Sample. J Rheumatol. 2019 Sept 01;46(9):1134–40. doi: 10.3899/jrheum.170990 30988126

[4] Sloan M, Premkumar A, Sheth NP. Projected volume of primary total joint arthroplasty in the U.S., 2014 to 2030. J Bone Joint Surg Am. 2018 Sept 05;100(17):1455–60. doi: 10.2106/JBJS.17.01617 30180053

[5] Yanyan B, Kaiyuan C, Xiao C, Xisheng W. Reports and analysis of amount of hip and knee arthroplasty in China from 2011 to 2019. Chinese Journal of Orthopaedics. 2020;40(21):1453–60.

[6] Kehlet H. Fast-track hip and knee arthroplasty. Lancet. 2013 Jan 01;381(9878):1600–2. doi: 10.1016/S0140-6736(13)61003-X 23663938

[7] Lindberg-Larsen M, Petersen PB, Corap Y, Gromov K, Jørgensen CC, Kehlet H, et al. Fast-track revision knee arthroplasty. Knee. 2022 Jan 01;34:24–33. doi: 10.1016/j.knee.2021.09.001 34894588

[8] Jenny JY, Courtin C, Boisrenoult P, Chouteau J, Henky P, Schwartz C, et al. Fast-track procedures after primary total knee arthroplasty reduce hospital stay by unselected patients: a prospective national multi-centre study. Int Orthop. 2021 Jan 01;45(1):133–8. doi: 10.1007/s00264-020-04680-0 32601722

[9] Drosos GI, Kougioumtzis IE, Tottas S, Ververidis A, Chatzipapas C, Tripsianis G, et al. The results of a stepwise implementation of a fast-track program in total hip and knee replacement patients. J Orthop. 2020 Sept 01;21:100–8. doi: 10.1016/j.jor.2020.03.004 32255989 PMC7114635

[10] Cheung A, Fu H, Cheung MH, Chan W, Chan PK, Yan CH, et al. How well do elderly patients do after total knee arthroplasty in the era of fast-track surgery? Arthroplasty. 2020 Jun 22;2(1):16. doi: 10.1186/s42836-020-00037-5 35236442 PMC8796349

[11] Clarius M, Noth U. Fast-track hip and knee arthroplasty. Orthopade. 2020 Jan 01;49(4):289. 32221717 10.1007/s00132-020-03891-y

[12] Hjort JD, Rud K, Kehlet H, Egerod I. Standardising fast-track surgical nursing care in Denmark. Br J Nurs. 2014 May 08;23(9):471–6. doi: 10.12968/bjon.2014.23.9.471 24820811

[13] Mabire C, Bachnick S, Ausserhofer D, Simon M. Patient readiness for hospital discharge and its relationship to discharge preparation and structural factors: A cross-sectional study. Int J Nurs Stud. 2019 Feb 01;90:13–20. doi: 10.1016/j.ijnurstu.2018.09.016 30522054

[14] Wang M, Lv L, Yu Z, Gao L, Lu Q, Ou J, et al. A cross-sectional study of readiness for discharge, chronic illness resources and post-discharge outcomes in patients with diabetic foot ulcer. Nurs Open. 2021 Sept 01;8(5):2645–54. doi: 10.1002/nop2.813 33730433 PMC8363364

[15] Weiss ME, Yakusheva O, Bobay KL, Costa L, Hughes RG, Nuccio S, et al. Effect of implementing discharge readiness assessment in adult medical-surgical units on 30-Day return to hospital: the READI randomized clinical trial. Jama Netw Open. 2019 Jan 04;2(1):e187387. doi: 10.1001/jamanetworkopen.2018.7387 30681712 PMC6484543

[16] Kaya S, Sain GG, Aydan S, Kar A, Teleş M, Yıldız A, et al. Patients’ readiness for discharge: predictors and effects on unplanned readmissions, emergency department visits and death. J Nurs Manage. 2018 Sept 01;26(6):707–16. doi: 10.1111/jonm.12605 29573007

[17] Coffey A, McCarthy GM. Older people’s perception of their readiness for discharge and post discharge use of community support and services. Int J Older People N. 2013 May 01;8(2):104–15. doi: 10.1111/j.1748-3743.2012.00316.x 22309350

[18] Pringle J, Drummond J, McLafferty E, Hendry C. Interpretative phenomenological analysis: a discussion and critique. Nurse Res. 2011 Jan 20;18(3):20–4. doi: 10.7748/nr2011.04.18.3.20.c8459 21560922

[19] Miller RM, Chan CD, Farmer LB. Interpretative phenomenological analysis: A contemporary qualitative approach. Couns Educ Superv. 2018 Jan 01;57(4):240–54.

[20] Tong A, Sainsbury P, Craig J. Consolidated criteria for reporting qualitative research (COREQ): a 32-item checklist for interviews and focus groups. Int J Qual Health C. 2007 Dec 01;19(6):349–57. doi: 10.1093/intqhc/mzm042 17872937

[21] Malterud K, Siersma VD, Guassora AD. Sample size in qualitative interview studies: guided by information power. Qual Health Res. 2016 Nov 01;26(13):1753–60. doi: 10.1177/1049732315617444 26613970

[22] Morse JM. "Data were saturated. …" Qual Health Res. 2015 May 01;25(5):587–8. doi: 10.1177/1049732315576699 25829508

[23] Walsh D, Downe S. Meta-synthesis method for qualitative research: a literature review. J Adv Nurs. 2005 Apr 01;50(2):204–11. doi: 10.1111/j.1365-2648.2005.03380.x 15788085

[24] Graneheim UH, Lundman B. Qualitative content analysis in nursing research: concepts, procedures and measures to achieve trustworthiness. Nurs Educ Today. 2004 Feb 01;24(2):105–12. doi: 10.1016/j.nedt.2003.10.001 14769454

[25] Høvik LH, Aglen B, Husby VS. Patient experience with early discharge after total knee arthroplasty: a focus group study. Scand J Caring Sci. 2018 Jun 01;32(2):833–42. doi: 10.1111/scs.12514 28833302

[26] Chen E, Brownell S, DiBrita T, Green A, McPherson L, Ragos R, et al. Patient perspectives on transitions from acute to community-based physiotherapy care following total knee replacement surgery within the context of a bundled care model. Physiother Can. 2023 May 01;75(2):190–7. doi: 10.3138/ptc-2021-0053 37736383 PMC10510550

[27] Büttner M, Mayer AM, Büchler B, Betz U, Drees P, Susanne S. Economic analyses of fast-track total hip and knee arthroplasty: a systematic review. Eur J Orthop Surg Tr. 2020 Jan 01;30(1):67–74. doi: 10.1007/s00590-019-02540-1 31512045

[28] Jansen JA, Kruidenier J, Spek B, Snoeker B. A cost-effectiveness analysis after implementation of a fast-track protocol for total knee arthroplasty. Knee. 2020 Mar 01;27(2):451–8. doi: 10.1016/j.knee.2019.09.014 31982250

[29] Woolhead GM, Donovan JL, Dieppe PA. Outcomes of total knee replacement: a qualitative study. Rheumatology. 2005 Aug 01;44(8):1032–7. doi: 10.1093/rheumatology/keh674 15870149

[30] Muhamad H, Yusoff M, Shokri AA, Sulaiman Z, Bakar RS, Zain NM. The Needs of orthopaedic patients in discharge planning. Malays Orthop J. 2022 Nov 01;16(3):36–43. doi: 10.5704/MOJ.2211.007 36589375 PMC9791901

[31] Weiss M, Yakusheva O, Bobay K. Nurse and patient perceptions of discharge readiness in relation to post discharge utilization. Med Care. 2010 May 01;48(5):482–6. doi: 10.1097/MLR.0b013e3181d5feae 20393364

[32] Epstein RM, Street RJ. The values and value of patient-centered care. Ann Fam Med. 2011 Mar 01;9(2):100–3. doi: 10.1370/afm.1239 21403134 PMC3056855

[33] Rognan SE, Kälvemark SS, Bengtsson K, Lie HB, Andersson Y, Mowé M, et al. Discharge processes and medicines communication from the patient perspective: A qualitative study at an internal medicines ward in Norway. Health Expect. 2021 Jun 01;24(3):892–904. doi: 10.1111/hex.13232 33761170 PMC8235877

[34] Wainwright TW, Memtsoudis SG, Kehlet H. Fast-track hip and knee arthroplasty…how fast? Brit J Anaesth. 2021 Feb 01;126(2):348–9. doi: 10.1016/j.bja.2020.09.038 33478683

[35] Burger M, Watson F, van Wyk A. A diarized journey: an interpretative phenomenological analysis of the older person’s lived experience of a hip or knee replacement within a fast-track programme. Bmc Geriatr. 2023 Sept 25;23(1):592. doi: 10.1186/s12877-023-04276-4 37743501 PMC10518952

[36] McKeown R, Kearney RS, Liew ZH, Ellard DR. Patient experiences of an ankle fracture and the most important factors in their recovery: a qualitative interview study. Bmj Open. 2020 Feb 04;10(2):e33539. doi: 10.1136/bmjopen-2019-033539 32024789 PMC7044932

[37] Nyborg I, Danbolt LJ, Kirkevold M. User participation is a family matter: A multiple case study of the experiences of older, hospitalised people and their relatives. J Clin Nurs. 2017 Dec 01;26(23–24):4353–63. doi: 10.1111/jocn.13765 28231606

[38] Solan LG, Beck AF, Brunswick SA, Sauers HS, Wade-Murphy S, Simmons JM, et al. The family perspective on hospital to home transitions: A qualitative study. Pediatrics. 2015 Dec 01;136(6):e1539–49. doi: 10.1542/peds.2015-2098 26620060

[39] Specht K, Kjaersgaard-Andersen P, Pedersen BD. Patient experience in fast-track hip and knee arthroplasty-a qualitative study. J Clin Nurs. 2016 Mar 01;25(5–6):836–45. doi: 10.1111/jocn.13121 26708610

[40] Wray CM, Jones CD. Bridging the Know-Do Gap in Hospital Care Transitions. Jama Intern Med. 2023 May 01;183(5):424–5. doi: 10.1001/jamainternmed.2023.0069 36939664

[41] Wang L, Lu H, Dong X, Huang X, Li B, Wan Q, et al. The effect of nurse staffing on patient-safety outcomes: A cross-sectional survey. J Nurs Manage. 2020 Oct 01;28(7):1758–66. doi: 10.1111/jonm.13138 32853457

[42] Jones KC, Austad K, Silver S, Cordova-Ramos EG, Fantasia KL, Perez DC, et al. Patient perspectives of the hospital discharge process: A qualitative study. J Patient Exp. 2023 Jan 20;10:672680940. doi: 10.1177/23743735231171564 37151607 PMC10159238

[43] Smith H, Grindey C, Hague I, Newbould L, Brown L, Clegg A, et al. Reducing delayed transfer of care in older people: A qualitative study of barriers and facilitators to shorter hospital stays. Health Expect. 2022 Dec 01;25(6):2628–44. doi: 10.1111/hex.13588 36193616 PMC9700150

[44] Piccenna L, Lannin NA, Gruen R, Pattuwage L, Bragge P. The experience of discharge for patients with an acquired brain injury from the inpatient to the community setting: A qualitative review. Brain Injury. 2016 Jan 20;30(3):241–51. doi: 10.3109/02699052.2015.1113569 26890803

[45] Gualandi R, Masella C, Piredda M, Ercoli M, Tartaglini D. What does the patient have to say? Valuing the patient experience to improve the patient journey. Bmc Health Serv Res. 2021 Apr 15;21(1):347. doi: 10.1186/s12913-021-06341-3 33858405 PMC8048032

