# Supplementary material for: Are patients ready for discharge from the hospital after fast-track total knee arthroplasty?-A qualitative study
Source: PLoS One. 2024 May 29;19(5):e0303935. doi: 10.1371/journal.pone.0303935 (PMC11135671; doi:10.1371/journal.pone.0303935)
Supplement: S1 File — (DOCX) [file pone.0303935.s002.docx]

**S1 File. Details of the fast-track clinical components**

**Are patients ready to be discharged from the hospital after fast-track total knee arthroplasty? -A qualitative study**

**-Patient education by attending physicians and nurses**

1) Assess patients for various complications

2) Ensure that a competent family caregiver is available

3) Use electronic imaging to visualize disease characteristics, treatment goals, implications, and plans

4) Establish discharge criteria in advance and inform patients that they can be discharged if they meet the criteria

**-Diet**

1) Solid food may be eaten 6 hours before surgery, and fluid food containing a small amount of sugar but no ethanol may be eaten 2 hours before surgery.

2) Take 40 ml of glucose solution with a concentration of 0.28 mol/L orally before entering the operating room.

3) To prevent nausea and vomiting, 10mg of dexamethasone sodium phosphate can be injected intravenously before the operation, and 10mg of dexamethasone sodium phosphate can be injected intravenously 6h after the operation and on the next day; 5mg of mosapride citrate tablets can be taken orally 3h before the operation, and 5mg/times after the operation, 3 times/d.

4) Fully awake after surgery, no nausea and vomiting can drink water, no adverse reactions and choking and coughing gradually after eating, do not do fasting and water fasting medical advice

**-Hyperalgesia and multimodal analgesia**

1) Oral celecoxib capsules 200 mg once daily starting 2 days before surgery

2) Preoperative intravenous dexamethasone sodium phosphate 10 mg.

3) Postoperative oral celecoxib capsule 200 mg twice daily

4) Postoperative oral tramadol hydrochloride tablets 50 mg twice daily

**-Functional training**

1) Preoperative contraction training of lung function, quadriceps muscle of lower limb, Tsukiji rope muscle and other functions under the guidance of professional rehabilitation physician, mastering the main points of movement training.

2) Postoperative 2-4 h knee extension and flexion, straight leg raising exercise, the first postoperative day out of bed activities, after discharge from the hospital to encourage isometric contraction of quadriceps, bilateral ankle pump exercise, knee mobility exercise.

3) Prevention of deep vein thrombosis of the lower limbs, family members urge patients to train rehabilitation movements.

**-Urinary voiding care**

1) All patients in this group were arranged to empty urine before anesthesia, and no catheter was left in place to prevent the risk of urethral injury and urinary tract infection after the catheter was left in place.

**-Blood management**

1) Some patients with malnutrition, anemia, and preoperative hemoglobin <120g/L were injected subcutaneously with erythropoietin injection 100IU/kg 1-3 times.

2) Tranexamic acid injection (15 mg/kg) dissolved in 100 ml of physiological saline for more than 10 min before skin incision.

3) Before closing the incision, soak the incision area with 3 g of tranexamic acid dissolved in 100 ml of physiological saline.

**-Infusion management**

1) Some patients have different degrees of cardiac insufficiency, and the central venous pressure should be monitored during the operation to avoid excessive fluid infusion.

2) Some patients are often combined with chronic diseases and have a strong reaction to the input of large quantities of low-temperature fluids. Postoperative infusion adopts infusion thermostat to keep the fluid temperature constant at 36.5-37.5℃, which is conducive to maintaining the patient's intraoperative constant temperature.

**-Discharge preparation**

1) Make a home rehabilitation training program for the patient, and the medical staff will follow up regularly by phone.

2) Come to outpatient clinic for review at 2 weeks, 1 month, 2 months and 3 months after operation.
