# Supplementary material for: Are patients ready for discharge from the hospital after fast-track total knee arthroplasty?-A qualitative study
Source: PLoS One. 2024 May 29;19(5):e0303935. doi: 10.1371/journal.pone.0303935 (PMC11135671; doi:10.1371/journal.pone.0303935)
Supplement: S2 File — (DOCX) [file pone.0303935.s003.docx]

**S2 File. Discharge Information for Patients**

**Are patients ready to be discharged from the hospital after fast-track total knee arthroplasty? -A qualitative study**

**Pre-operative informing of discharge criteria.**

1) Adequate pain control (VAS <5).

2) Independence in personal care: getting in and out of bed, getting up from a chair, sitting down, going to the bathroom, walking 70 meters (the length of our corridor) with an appropriate walker, TKA flexion criteria (>90 degrees).

3) Patient accepts to discharge himself/herself.

**Post-operative Discharge Targeted Education**

**Functional exercises**

1) Weight-bearing straight leg raising, place a 1-pound sandbag at the ankle joint of the affected limb, with the leg at 70 degrees from the bed, and then do straight leg raising exercises, practice 3 times a day, and then gradually increase the sandbag to 5 pounds.

2) Joint mobility (flexion and extension) exercises: each exercise seeks to improve the angle, if the flexion and extension angle for a long time (> two weeks) without progress, there is a possibility of joint adhesion, so it should be highly valued, adhere to the exercise.

3) Squatting exercises with objects: patients hold objects with both hands, flatten the waist and do squatting exercises.

**Proper diet**

1. In principle, give high protein, high vitamin diet, eat more calcium-rich foods to prevent osteoporosis.

2) Three meals a day can be supplemented with calcium milk or calcium-fortified yogurt, and more fish floss, shrimp skin, shrimp, soy products and other foods.

**Medication instructions**

1. If the pain worsens and interferes with sleep at night, chondroitin sulfate sodium tablets and loxoprofen sodium tablets can be taken orally for anti-inflammatory and analgesic purposes.
2. If improper postoperative care leads to infection, use clindamycin hydrochloride for injection to fight infection as prescribed by the doctor.
3. Deep vein thrombosis (DVT) prophylaxis education: use low molecular heparin calcium injection as prescribed by the doctor, or oral rivaroxaban, aspirin and other drugs.

**Appropriate home set-up and competent adult caregivers**

1. Avoid living in damp and cold environment.
2. Notify the time of review. If there are unexplained fever symptoms, severe pain in the affected knee joint or sudden shortening of the affected limb, seek medical attention promptly.

**Instructions for daily living**

1. Pay attention to personal hygiene, keep the wound dry after bathing, and don't apply ointments or salves on the wound.
2. Avoid wearing tight pants to reduce friction.
3. Maintain ideal body weight to reduce the burden on the knee joint. If the body mass index (BMI) is greater than 24, you need to lose weight.
4. With increased activity, joint pain, swelling and stiffness may occur in the morning, which are normal.
5. You can do some daily chores, but avoid overloading the knee joints, such as lifting heavy objects and going up and down stairs.
6. Avoid walking on uneven or smooth surfaces to avoid falling;
7. Avoid squatting, climbing, strenuous running and jumping, long-distance walking and other behaviors. After 6 months, swim, ride bicycle and resume normal activities.
