# Supplementary material for: Are patients ready for discharge from the hospital after fast-track total knee arthroplasty?-A qualitative study
Source: PLoS One. 2024 May 29;19(5):e0303935. doi: 10.1371/journal.pone.0303935 (PMC11135671; doi:10.1371/journal.pone.0303935)
Supplement: S3 File — (DOCX) [file pone.0303935.s004.docx]

**S3 File. Patients interview guide.**

**Are patients ready to be discharged from the hospital after fast-track total knee arthroplasty? -A qualitative study**

1. **Please tell me about your recent experience about fast-track total knee arthroplasty**
   1. May I ask how many days since your surgery?
   2. Are you satisfied with the whole procedure of your previous total knee arthroplasty?
   3. Can you tell me briefly what's uncomfortable in these days after the operation?
2. **Please tell me what you remember about the discharge process from the time your care team gave you the order to leave the hospital until now.**
   1. What hospital staff explained the discharge plan to you? How familiar were you with this person and did they know what had been going on with you in the hospital?
   2. When did they first discuss your discharge plan with you?
   3. Did you have questions? Were they answered?
   4. Were you alone when you had your discharge planning discussion with hospital staff or did you have a friend/family member with you?
3. **Are you satisfied with the entire process of preparing for discharge care services after total knee replacement?**
   1. If it's satisfactory, can you be more specific?
   2. If not, can you be more specific?

e.g., Whether the nurse respects your religion and values, whether your privacy is protected, whether to listen to your concerns, whether they are eager to help, whether the instructions for self-care are appropriate, demonstrate the correct behaviour on the spot, the timing of the message is appropriate, consistency of message...

1. **Are there any medical treatment factors associated with post-total knee arthroplasty that have not been discussed in the later stages of discharge preparation?**

e.g., pain management, surgical wound treatment, respiratory therapy, exercise rehabilitation, proper medication and functional exercise...

1. **How do you feel about your post-op recovery so far?**
   1. Can you describe the physical strength of the day?

e.g., Stretching legs, raising thighs, bending knees, standing, walking, squatting...

- 1. Can you explain the extent of your self-care now?

e.g., handling wound dressings, dressing changes, exercise, rehabilitation, hygiene, bathing, walking, toileting, eating, taking the right medication at the right time...

- 1. Can you describe your level of knee pain at rest, after simple bedside activities, and during sleep at night, respectively?

1. **Your doctor has given you discharge instructions for now, do you feel you are ready to be discharged?**
   1. Get ready. What do you think went well?
   2. Not yet. What do you think could have been done better?

e.g., Financial support, adaptability, family-social support utilisation, rehabilitation self-efficacy, disease Control, pain management, daily living considerations (hygiene, bathing, walking, toileting, eating, medication, observations, community services or counselling, etc.)...

- 1. Currently unknown.

1. **Can you share your inner feelings now that you've heard you're going to be discharged from the hospital?**
   1. Are you confident in the discharge process and the care team you worked with?
   2. Are you confident in your knowledge of disease self-care, related medical knowledge, considerations, and next steps in your treatment plan?
   3. Are you confident in adhering to the completion of functional exercises, personal care and follow-up treatment?
2. **What would you like the nursing staff to do to provide you pre- and post-discharge?**
   1. pre-discharge

e.g., Risk factors, pain management, complications, discharge referrals, discharge reimbursement contributions, discharge with medication...

- 1. post-discharge

e.g., Scheduled telephone follow-up visits by healthcare professionals, linkage with community or lower level hospitals, outpatient review...

1. **If you are making recommendations to the hospital to improve the discharge process, what would you recommend?**
   1. What should stay the same?
   2. What should be done differently?
2. **Do you feel like the hospital staff took your opinions and preferences into account when helping to plan your discharge?**
   1. What do you feel like they really considered your opinion on?
   2. Did you feel like your voice was heard?
   3. What preferences did you feel were not considered?
3. **Can you get help from family members, neighbours, friends, colleagues, patients?**
   1. Do they know what had been going on with you in the hospital?
   2. Do they support you when you're in trouble?

e.g., Financial support, emotional regulation, housework, personal care, supervision of rehabilitation exercises

- 1. Were you alone when you had your discharge planning discussion with hospital staff or did you have a friend/family member with you?

**Thank you for your cooperation!**
