# Supplementary material for: Are patients ready for discharge from the hospital after fast-track total knee arthroplasty?-A qualitative study
Source: PLoS One. 2024 May 29;19(5):e0303935. doi: 10.1371/journal.pone.0303935 (PMC11135671; doi:10.1371/journal.pone.0303935)
Supplement: S1 Table — (DOCX) [file pone.0303935.s005.docx]

**S1 Table. The structural analysis and how the themes and subthemes emerged.**

| **Meaningful Statements** | **meaningful unit** | **Sub-themes** | **Themes** |
| --- | --- | --- | --- |
| *“****The surgery went well...*** *I feel way stronger than before. I used to struggle with stairs, and although my knee hurts a bit now, it's manageable, and I'm recovering nicely... I can even make it to the bathroom on my own...****Getting home is what I'm really looking forward to.”*** *(*ZTA*, Male, 66 years old, Post-op day 3)*  ***Prep started right after the surgery...*** *They had me bending my knees, stretching my legs in bed, and even practicing with a walker... Those walks kept getting longer, a little tired, but the docs and nurses were really on top of things...* ***I'm all set to head home now."*** *(ZYX,Female, 54 years old, Post-op day 2)*  *(Day 2 post-op) I felt thankful for* ***my surgeon*** *who* ***told me*** *during the check-up* ***that my test results were good, and my rehab exercises were on track...*** *They said* ***I'd be going home soon, and I've been eagerly waiting for that moment.****” (GWX, Male, 57 years old, Post-op day 3)*  *The whole process of the surgery went well, I've been in and out of the hospital for over a week now,* ***today is the third day after the surgery and I feel like I'm healing day by day,*** *now I just need to be patient and wait for the doctor's approval to be discharged. (ZC, Male, 68 years old, Post-op day 4)*  ***The doctor said that if I could easily bend (90°) and straighten (180°) my knee today, I would be out of the hospital soon.*** *I know what the doctor expects from me ..... So I am extra excited and working hard. (LW, Male, 69 years old, Post-op day 4)* | ***The surgery went well..Getting home is what I'm really looking forward to.***  ***Prep started right after the surgery...I'm all set to head home now."***  ***my surgeon told me that my test results were good, and my rehab exercises were on track...***  ***I'd be going home soon, and I've been eagerly waiting for that moment.***  ***today is the third day after the surgery and I feel like I'm healing day by day,***  ***The doctor said that if I could easily bend (90°) and straighten (180°) my knee today, I would be out of the hospital soon.*** | Satisfaction with improved functioning and a sense of reborn joy | Preparing for discharge despite concerns about symptoms |
| ***It happened so quickly that I didn't even realize*** *I was suddenly kicked out of the hospital, and now I feel like I'm just winging it...* ***If only they had let me stay one more day, I think I could have absorbed all this info...*** *(SXQ, Female, 69 years old, Post-op day 3)*  *Now it's all up to me...* ***There are still plenty of things I'm uncertain about...*** *Like how I'm gonna manage a shower when I get back... And standing for a while doesn't feel quite right, 'cause the skin on this leg is numb and doesn't quite feel like mine, but maybe that's just part of the deal. (WTY, Female, 61 years old, Post-op day 2)*  *Discharged. It's a mess. Quickly....* ***I'm not so sure I would have done subsequent rehab movements on my own*** *without them (doctors, rehabbers, nurses...),* ***A sense of being left to its own devices.****(DJ, Female ,71 years old, Post-op day 3)* | ***It happened so quickly that I didn't even realize...If only they had let me stay one more day, I think I could have absorbed all this info...***  ***There are still plenty of things I'm uncertain about...***  ***I'm not so sure I would have done subsequent rehab movements on my own...A sense of being left to its own devices.*** | Feeling of helplessness |  |
| *I don't wanna go back and feel like folks are giving me the side-eye 'cause of this knee replacement.* ***In the hospital, we're all in the same boat, and there's no judgment like that...*** *But the doc told me to head back as soon as possible, and it felt like they were just gonna drop us to make room for new patients. (CBC, Female, 78 years old, Post-op day 3)*  *I'm not sure if folks will treat me differently when I return because of this artificial knee.* ***They might think I'm in the same boat as someone with a disability.*** *(ZYX, Female, 54 years old, Post-op day 2)* | ***In the hospital, we're all in the same boat, and there's no judgment like that（discriminated against）***  ***They might think I'm in the same boat as someone with a disability.*** | Stigmatisation |  |
| ***Can I really go for a deep squat? Could it possibly mess up this fake knee joint? I'm not entirely convinced this artificial joint is as great as the doc claims it to be.*** *(DJ, Female ,71 years old, Post-op day 3)*  *The guy in the bed next to mine had his surgery a day before me, and he got sent home yesterday. I'm a bit worried that the new joint isn't as good as the old one, and* ***I'm kinda scared that if I push it too hard, I might end up needing another replacement...*** *But I guess it's probably my turn to go home too. (ZYJ, Female, 63 years old, Post-op day 3)* | ***Can I really go for a deep squat? Could it possibly mess up this fake knee joint? I'm not entirely convinced this artificial joint is as great as the doc claims it to be.***  ***I'm kinda scared that if I push it too hard, I might end up needing another replacement...*** | Anxiety about prosthetic function |  |
| ***I'm feeling great and totally ready to keep up with the exercises, even if it means dealing with some intense pain while at home this time.*** *(YMY, Female, 65 years old, Post-op day 2)*  ***This surgery is a big deal in my life, I'm absolutely committed to my post-surgery rehab...****Just yesterday, I managed to fully straighten my leg (up to 180°), and that took a lot of self-discipline with all those little exercises.* ***I'm confident I can keep up with the rehab routine I've been taught.*** *(GXP, Female, 68 years old, Post-op day 3)*  *This time around, I didn't just shell out a bunch of cash for the surgery; I was also dead-set on giving my knee the proper care it deserves...* ***Post-op rehab is a big deal, and I can't afford to lose in this battle.*** *(YM, Male, 74 years old, Post-op day 3)* | ***ready to keep up with the exercises, even if it means dealing with some intense pain while at home this time.***  ***This surgery is a big deal in my life, I'm absolutely committed to my post-surgery rehab.***  ***I'm confident I can keep up with the rehab routine I've been taught.***  ***Post-op rehab is a big deal, and I can't afford to lose in this battle.*** | Highly motivated and currently on the road to recovery. | Managing the rehabilitation difficulties |
| *Well rested is important,* ***the first night after surgery, I didn't really get into a deep sleep and the pain got worse.*** *Yesterday morning, I washed up, but it wore me out(1-2 metres). I'd like to walk around more, but honestly, it's quite a struggle. Sometimes, pushing it can be more of a burden. (XZZ, Female, 68 years old, Post-op day 3)*  *I barely noticed the minor pains, but over the last three days, the painkillers just weren't cutting it. I had to hobble around the room with crutches,* ***and I'll admit, I was feeling pretty scared. Then the GP showed up and gave me an extra dose of morphine...*** *and I was cautioned to take it easy with my exercises. It was tough to dive into the rehab routine right away, and I felt a bit overwhelmed. (ZWQ, Female,73 years old, Post-op day 4)*  *I was going to listen to my doctor and take the pills,* ***but I think painkillers are always a chemical that may harm my body in a subtle way*** *..... It's better to tolerate the pain without medication...if I can.(LYQ, Male, 56 years old, Post-op day 2)* | ***I didn't really get into a deep sleep and the pain got worse.***  ***I think painkillers are always a chemical that may harm my body in a subtle way***  ***I'll admit, I was feeling pretty scared. Then the GP showed up and gave me an extra dose of morphine...*** | Medication management |  |
| *The doctor said it's best to keep the wound out of water for now,* ***but I can only bathe in the shower at home, so I may have to give up bathing for a while.****(ZTA, Male, 66 years old, Post-op day 3)*  ***I mean... it's just not as sterile at home as it is in a hospital,*** *you know? In the hospital,* ***there's a strict no-smoking policy, and everything around you is equipped with handles and gets sanitized regularly.*** *But at home, it can be kind of germ-central, with no handles in the bathroom, and I'm always worried about tripping or getting some nasty infection when I'm moving around. (DJ, Female, 71 years old, Post-op day 3)*  *That walker won't fit in our bathroom at home, and I'm worried I might take a tumble without it. It'd be great to have some kind of handrail I can grab onto when I'm moving around and getting up from the toilet. (JH, Female, 53 years old, Post-op day 2)*  ***My doc and nurse told me to stick to a high-protein and light diet****, and they kept a close eye on what I was eating in the hospital. But you know,* ***I'm a fan of spicy food myself, and my family loves throwing chili peppers into our cooking.*** *That's just how we roll when it comes to eating habits. (XLE, Male, 62 years old, Post-op day 3)* | ***I can only bathe in the shower at home, so I may have to give up bathing for a while.***  ***it's just not as sterile at home as it is in a hospital,***  ***there's a strict no-smoking policy, and everything around you is equipped with handles.***  ***My doc and nurse told me to stick to a high-protein and light diet. I'm a fan of spicy food myself, and my family loves throwing chili peppers into our cooking.*** | Environmental change |  |
| *I really appreciated the calls and messages of support from my friends when they heard about my major surgery this time, but* ***I won't be attending too many gatherings because I hardly go out on my own anymore.*** *(YS, Male, 67 years old, Post-op day 3)*  ***My husband... he actually tried not to share the same bed with me... he was worried about accidentally hurting my wounds during the night... Sometimes, it does get a bit lonely.*** *(XGB, Female, 69 years old, Post-op day 3)*  ***My son burned through all his saved-up vacation days.*** *Truth be told, he didn't really know how to help me get up or out of bed. He even made me a cup of tea, which I can't drink right now... But turning him down would feel impolite,* ***especially since he's given up his precious time and spent a lot of money.*** *(LW, Male, 69 years old, Post-op day 4)*  *My partner died a couple of years ago...****my daughter was busy with work*** *so she hired a domestic helper to look after me, a stranger to me... but* ***she put a lot of effort into this.****(LYQ, Male, 56 years old, Post-op day 2)* | ***I won't be attending too many gatherings because I hardly go out on my own anymore.***  ***My husband...tried not to share the same bed with me... worried about accidentally hurting my wounds during the night...Sometimes, it does get a bit lonely.***  ***My son burned through all his saved-up vacation days. especially since he's given up his precious time and spent a lot of money.***  ***my daughter was busy with work...but she put a lot of effort into this*** | Strained social and intimate relationships |  |
| ***They just kept repeating things in case I forgot, treating me like one of their own family.*** *They were always concerned whenever I got an injection or a change in medication... They were with me from the surgery all the way to when I was ready to leave, and I really felt like I could trust this team. (GYH, Female, 80 years old, Post-op day 5)*  ***They actually came right to our bedsides to show us how to move, taught us by demonstrating with their own bodies****, and made sure we fully understood. They were super patient, used really clear language, and avoided using overly technical terms. (YM, Male, 74 years old, Post-op day 3)*  ***The nurses did teaching right at our bedsides and constantly shared success stories of other patients... that really boosted my confidence.*** *(GWX, Male, 57 years old, Post-op day 3)* | ***They just kept repeating things in case I forgot, treating me like one of their own family.***  ***They(nurses) actually came right to our bedsides to show us how to move, taught us by demonstrating with their own bodies***  ***The nurses did teaching right at our bedsides and constantly shared success stories of other patients... that really boosted my confidence.*** | The caring bedside manner of the nursing staff | Creating conditions for safe transition |
| *Later that morning, they said they'd be back in an hour or two to switch up my meds, and the rehab person was scheduled to come by at 10:00 a.m.* ***I didn't want to start rehab before the med change because I knew it would make the pain worse. The nurse understood my concern and shifted the med change schedule, which I really appreciated.*** *(XGB, Female,69 years old, Post-op day 3)*  ***The nurses handed us a bunch of booklets loaded with information, but I had a hard time with them because I've got presbyopia,*** *and those paper booklets looked all blurry to me. So, when I asked the nurses about it, they hooked me up with a bunch of rehab videos. (ZTA, Male, 66 years old, Post-op day 3)*  *The day after my surgery,* ***I realized that the benches in the ward were way too low for me, probably because of my height (187 cm)****, and with the surgery, it was a struggle to sit down.* ***I mentioned this to the nurse, and she somehow managed to find me a taller chair.*** *(XLE, Male, 62 years old, Post-op day 3)* | ***I didn't want to start rehab before the med change because I knew it would make the pain worse. The nurse understood my concern and shifted the med change schedule, which I really appreciated.***  ***The nurses handed us a bunch of booklets, but I’ve got presbyopia. So, they hooked me up with a bunch of rehab videos...***  ***The benches in the ward were way too low for me, probably because of my height (187 cm)...I mentioned this to the nurse, and she somehow managed to find me a taller chair.*** | Listening to patients |  |
| *I'll be getting out of here soon, and I know the nurses are swamped with their to-do list. I'm still feeling a bit unsure about going home all of a sudden.* ***I was wondering if I could have your contact info because you know my health situation better than anyone else****. (SXQ, Female,69 years old, Post-op day 3)*  *I'm constantly feeling swamped by the sheer amount of info and what's coming up next. I'm worried it might be too much to handle on my own. Thankfully,* ***the doc's gone ahead and created a WeChat group for us.*** *It's a place where we can chat and swap stories,* ***which honestly gives me some peace of mind.*** *(LYQ, Male, 56 years old, Post-op day 2)*  *My primary care doctor said...****I am already severely overweight and suggested I could be referred to a bariatric centre for treatment as a next step****, and I would like to know what to do. (ZWQ, Female,73 years old, Post-op day 4)* | ***I was wondering if I could have your contact info because you know my health situation better than anyone else****.*  ***The doc's gone ahead and created a WeChat group for us, which honestly gives me some peace of mind.***  ***I am already severely overweight and suggested I could be referred to a bariatric centre for treatment as a next step*** | A humanised continuing care and referral services |  |
