# Supplementary material for: Are patients ready for discharge from the hospital after fast-track total knee arthroplasty?-A qualitative study
Source: PLoS One. 2024 May 29;19(5):e0303935. doi: 10.1371/journal.pone.0303935 (PMC11135671; doi:10.1371/journal.pone.0303935)
Supplement: Attachment — Submitted filename: Response to Reviewers.docm [file pone.0303935.s006.docm]

Robert Jeenchen Chen, PhD

Academic Editor

PLOS ONE

Re:	PONE-D-23-33287

Are patients ready for discharge from the hospital after fast-track total knee arthroplasty? -A qualitative study

**Additional editor comments**

Thank you for submitting your manuscript to PLOS ONE. After careful consideration, we feel that it has merit but does not fully meet PLOS ONE’s publication criteria as it currently stands. Therefore, we invite you to submit a revised version of the manuscript that addresses the points raised during the review process.

**Author response:** Thank you for the opportunity to submit a revised version of the manuscript. We greatly appreciate the reviewers’ comments and consider addressing these have led to an improved manuscript. We can confirm the manuscript meets PLOS ONE's style requirements, including those for file naming. Please find our response to the reviewers’ comments below. We have also included the manuscript changes and used red type below to highlight the relevant changes to the manuscript text.

**Reviewer #1**

**Reviewer #1:** It is not clear what this study adds to existing knowledge. Please better justify the present study taking into account existing literature.

https://pubmed.ncbi.nlm.nih.gov/26708610/

https://pubmed.ncbi.nlm.nih.gov/31769559/

https://pubmed.ncbi.nlm.nih.gov/30297138/

https://pubmed.ncbi.nlm.nih.gov/31227003/

<https://pubmed.ncbi.nlm.nih.gov/27345939/>

**Author response:** Thank you for your question and for providing us with articles that need to be re-read carefully for this purpose. Our response is as follows: First, a review of the literature found that current qualitative researches on this subject have focused primarily on the staged experiences of patients undergoing fast-track total knee/hip arthroplasty (TKA/THA) throughout the discharge process or in the short-term post-discharge period, and appeared to have limited understanding of patients' experiences at the time of receiving discharge instructions. The time point at this study was chosen to take place within 24 hours of receiving discharge instructions anticipating more feedback from patients as they face the decision to be discharged. Second, we recognized that this study did share similarities with the results of prior studies, such as poor pain management, uncertainty and lack of confidence in recovery due to poor information, stigma, and social relationships still exist. However, in contrast to previous studies, this study, in addition to revealing the challenges and shortcomings faced by patients, was fortunate to hear some positive responses from patients regarding clinical care practices, such as compassionate bedside manner, listening to the patient's voice, and humanized continuity of care and referral services. This also has implications for the continued maintenance and renewed efforts to improve discharge care services in the future.

**Author change:** It adds valuable knowledge about the understanding of how patients treated with TKA under fast track setting when facing discharge, in addition to revealing the challenges patients face, it also adds some positive responses about clinical care practices. (page 20, lines 374-377)

**Reviewer #1:** Line 86: What is the evidence of the benefits of patient involvement?

**Author response:** Thank you very much for your comments, and after reviewing the relevant literatures, we added the benefits of patient involvement in the revised manuscript, including reduced unplanned readmission and mortality rates, improved patient satisfaction, and also helping to optimize the discharge planning process. References have also been added to the revised manuscript.

**Author change:** Weiss(15) and Coffey(17) have advocated that incorporating patients' own perspectives in evaluating discharge readiness reduces unplanned readmission and mortality rates, as well as optimizing the discharge planning process while improving patient satisfaction. (page 4-5, lines 81-84)

**Reviewer #1:** Lines 95 to 100: the objective needs to be made clearer.

**Author response:** Thank you very much for your suggestion, and in the revised manuscript we have clarified the objectives of this study even more.

Actually, this study contains two explicit objectives. Objective 1: To describe the discharge preparation experiences of patients after fast-track TKA and to clarify whether patients are consciously prepared for discharge. Objective 2: To explore what gaps exist in the current discharge preparation care service for TKA under fast-track and what can be improved.

**Author change:** This study aims to describe patients' experiences of their discharge instructions following TKA under FTS in China to clarify whether patients consciously prepare for discharge. Additionally, this study attempts to explore what gaps exist in current discharge preparation care service and what could be improved. (page 5, lines 92-95)

**Reviewer #1:** Line 98: this goes beyond nursing and includes physicians and rehab specialists.

**Author response:** Many thanks for pointing this out. This does include not only the scope of nursing, but also physicians and rehabilitation specialists, and given the readership of the results of this study, we decided to remove this item to ensure that it falls within the scope of nursing responsibilities.

**Author change:** ~~The ultimate goal is to enhance health promotion efforts related to discharge preparation, thereby improving the overall patient experience and prognosis.~~ (page 5, lines 92-95)

**Reviewer #1:** Line 124: -More details are needed on TKA patient characteristics: due to OA only or other health problems such as fractures, RA, primary elective, etc? Were revisions and bilaterals included or not?

**Author response:** We added more details on patient characteristics.In this study, we only considered patients who underwent their first unilateral TKA for advanced osteoarthritis of the knee(KOA), excluding fractures, rheumatoid arthritis, or revisions. In addition, all patients we included for interview were primary elective.

**Author change:** The inclusion criteria were as follows: a) first unilateral TKA for advanced KOA; b) take primary elective surgery. c) receive fast-track system; d) receipt of a discharge order; e) demonstrate effective communication skills; and f) willingness to join the study and willingness to join the study and consent in writing. (page 6-7, lines 119-123)

**Reviewer #1:** Line 124: -Among these patients, describe how are patients selected for fast-track

**Author response:** Thank you very much for your suggestion. Patients recruitment for this study was from May 2023 to July 2023. From May 2023, all elective patients (ASA classification I, II and stable III) diagnosed with osteoarthritis of the knee and scheduled to undergo TKA were enrolled in the fast-track system.We have added this to the text as requested.

**Author change:** We purposively recruited patients who underwent fast-track TKA between May 2023 and July 2023 in the orthopaedic wards of a tertiary A-level hospital located in Jinan, Shandong Province, China. Patients with ASA classification of class I, II and stable III were enrolled in the fast-track system(S1 File). (page 6, lines 114-115)

**Reviewer #1:** Line 124: -Details of the fast-track clinical components are needed. It is not clear what this program consists of.

**Author response:** Thanks for your kind suggestions, which are valuable for improving the accuracy of the manuscript. In the revised manuscript, we have added details about the clinical components of the fast-track, which we have placed in the S1 File (Details of the fast-track clinical components) for your review.

**Author change:** Patients with ASA classification of class I, II and stable III were enrolled in the fast-track system (S1 File). (page 6, lines 114-115)

**Author change: S1 File. Details of the fast-track clinical components.**（page 32, line 641）

**Details of the fast-track clinical components**

-Patient education by attending physicians and nurses

1) Assess patients for various complications

2) Ensure that a competent family caregiver is available

3) Use electronic imaging to visualize disease characteristics, treatment goals, implications, and plans

4) Establish discharge criteria in advance and inform patients that they can be discharged if they meet the criteria

-Diet

1) Solid food may be eaten 6 hours before surgery, and fluid food containing a small amount of sugar but no ethanol may be eaten 2 hours before surgery.

2) Take 40 ml of glucose solution with a concentration of 0.28 mol/L orally before entering the operating room.

3) To prevent nausea and vomiting, 10mg of dexamethasone sodium phosphate can be injected intravenously before the operation, and 10mg of dexamethasone sodium phosphate can be injected intravenously 6h after the operation and on the next day; 5mg of mosapride citrate tablets can be taken orally 3h before the operation, and 5mg/times after the operation, 3 times/d.

4) Fully awake after surgery, no nausea and vomiting can drink water, no adverse reactions and choking and coughing gradually after eating, do not do fasting and water fasting medical advice

-Hyperalgesia and multimodal analgesia

1) Oral celecoxib capsules 200 mg once daily starting 2 days before surgery

2) Preoperative intravenous dexamethasone sodium phosphate 10 mg.

3) Postoperative oral celecoxib capsule 200 mg twice daily

4) Postoperative oral tramadol hydrochloride tablets 50 mg twice daily

-Functional training

1) Preoperative contraction training of lung function, quadriceps muscle of lower limb, Tsukiji rope muscle and other functions under the guidance of professional rehabilitation physician, mastering the main points of movement training.

2) Postoperative 2-4 h knee extension and flexion, straight leg raising exercise, the first postoperative day out of bed activities, after discharge from the hospital to encourage isometric contraction of quadriceps, bilateral ankle pump exercise, knee mobility exercise.

3) Prevention of deep vein thrombosis of the lower limbs, family members urge patients to train rehabilitation movements.

-Urinary voiding care

1) All patients in this group were arranged to empty urine before anesthesia, and no catheter was left in place to prevent the risk of urethral injury and urinary tract infection after the catheter was left in place.

-Blood management

1) Some patients with malnutrition, anemia, and preoperative hemoglobin <120g/L were injected subcutaneously with erythropoietin injection 100IU/kg 1-3 times.

2) Tranexamic acid injection (15 mg/kg) dissolved in 100 ml of physiological saline for more than 10 min before skin incision.

3) Before closing the incision, soak the incision area with 3 g of tranexamic acid dissolved in 100 ml of physiological saline.

-Infusion management

1) Some patients have different degrees of cardiac insufficiency, and the central venous pressure should be monitored during the operation to avoid excessive fluid infusion.

2) Some patients are often combined with chronic diseases and have a strong reaction to the input of large quantities of low-temperature fluids. Postoperative infusion adopts infusion thermostat to keep the fluid temperature constant at 36.5-37.5℃, which is conducive to maintaining the patient's intraoperative constant temperature.

-Discharge preparation

1) Make a home rehabilitation training program for the patient, and the medical staff will follow up regularly by phone.

2) Come to outpatient clinic for review at 2 weeks, 1 month, 2 months and 3 months after operation.

**Reviewer #1:** Line 138: who provided the training?

**Author response:** The theory of qualitative research was studied and assessed in the School of Nursing, and the internship hospital (Research Department of the First Affiliated Hospital of Shandong First Medical University) provided training on interviewing techniques.

**Author change:** Received extensive theoretical assessment on qualitative research from the School of Nursing and systematic training on interviewing skills from the First Affiliated Hospital of Shandong First Medical University. (Page 7, Lines 135-138)

**Reviewer #1:** Line 142: provide in detail what information is given to patients before and after surgery regarding discharge, both paper and orally (could be given in an appendix).

**Author response:** Thank you for your advice. Discharge information mainly includes: informing of discharge criteria, functional exercises, proper diet, medication instructions, appropriate home set-up and competent adult caregivers, and instructions for daily living. We have included this section in S2 File (Discharge Information for Patients) for your reference.

**Author change:** Patients were warmly invited to participate in interviews when the researcher (NL) received and verbally explained the discharge instructions (S2 File). (Page 7, Line 140)

**Author change: S2 File. Discharge Information for Patients.** (Page 32, Line 642)

**Discharge Information for Patients**

Pre-operative informing of discharge criteria.

1) Adequate pain control (VAS <5).

2) Independence in personal care: getting in and out of bed, getting up from a chair, sitting down, going to the bathroom, walking 70 meters (the length of our corridor) with an appropriate walker, TKA flexion criteria (>90 degrees).

3) Patient accepts to discharge himself/herself.

Post-operative Discharge Targeted Education

Functional exercises

1) Weight-bearing straight leg raising, place a 1-pound sandbag at the ankle joint of the affected limb, with the leg at 70 degrees from the bed, and then do straight leg raising exercises, practice 3 times a day, and then gradually increase the sandbag to 5 pounds.

2) Joint mobility (flexion and extension) exercises: each exercise seeks to improve the angle, if the flexion and extension angle for a long time (> two weeks) without progress, there is a possibility of joint adhesion, so it should be highly valued, adhere to the exercise.

3) Squatting exercises with objects: patients hold objects with both hands, flatten the waist and do squatting exercises.

Proper diet

1) In principle, give high protein, high vitamin diet, eat more calcium-rich foods to prevent osteoporosis.

2) Three meals a day can be supplemented with calcium milk or calcium-fortified yogurt, and more fish floss, shrimp skin, shrimp, soy products and other foods.

Medication instructions

1) If the pain worsens and interferes with sleep at night, chondroitin sulfate sodium tablets and loxoprofen sodium tablets can be taken orally for anti-inflammatory and analgesic purposes.
2) If improper postoperative care leads to infection, use clindamycin hydrochloride for injection to fight infection as prescribed by the doctor.
3) Deep vein thrombosis (DVT) prophylaxis education: use low molecular heparin calcium injection as prescribed by the doctor, or oral rivaroxaban, aspirin and other drugs.

Appropriate home set-up and competent adult caregivers

1) Avoid living in damp and cold environment.
2) Notify the time of review. If there are unexplained fever symptoms, severe pain in the affected knee joint or sudden shortening of the affected limb, seek medical attention promptly.

Instructions for daily living

1) Pay attention to personal hygiene, keep the wound dry after bathing, and don't apply ointments or salves on the wound.
2) Avoid wearing tight pants to reduce friction.
3) Maintain ideal body weight to reduce the burden on the knee joint. If the body mass index (BMI) is greater than 24, you need to lose weight.
4) With increased activity, joint pain, swelling and stiffness may occur in the morning, which are normal.
5) You can do some daily chores, but avoid overloading the knee joints, such as lifting heavy objects and going up and down stairs.
6) Avoid walking on uneven or smooth surfaces to avoid falling;
7) Avoid squatting, climbing, strenuous running and jumping, long-distance walking and other behaviors. After 6 months, swim, ride bicycle and resume normal activities.

**Reviewer #1:** Line 152: on how many patients?

**Author response:** This pilot study included a total of 3 patients.

**Author change:** To ensure construct validity in the qualitative interviews, a pilot study was conducted on three patients. (Page 8, Line 151)

**Reviewer #1:** Line 153: describe in detail how the guide was developed.

**Author response:** Thank you for your advice, and we have added specific details in the revised manuscript, which you are invited to review.

**Author change:** The final interview guide was co-developed by all the authors based on the research questions, and careful consideration was given to all issues in this area, which included the patient's surgical situation and outcome assessment, experience and assessment of discharge, and consideration of the patient's support level and dilemmas in terms of caregivers, family members, and social support, etc. To minimize the likelihood of missing any potentially relevant factors (e.g., pain management, surgical wound treatment, respiratory therapy, and proper medication were listed as medical factors affecting the later stages of discharge readiness) were pre-included to elicit more thought and response during the pilot study. The "yes or no" questions were further designed with open-ended questions (e.g., Do you feel ready for discharge? If not, what do you think needs to be done better?). (Page 8, Lines 151-162)

**Author change:** The complete interview guide is available in S3 File. (Page 32, Line 643)

**Reviewer #1:** Line 160: How was this taken into account in the analyses?

**Author response:** Thank you very much for asking this question, and our response is as follows:The interview questions involved probing patients' true feelings about their readiness for discharge, as well as patients' comments and suggestions about the quality of care they received on discharge, and although we explained to patients that we were not responsible for physical health assessments and quality of care checks within the unit, participants may have still reported what they thought the researchers would want to hear (this is a point we have already touched upon in the limitations of this study), and we preferred to hear patients' most honest expression of their views. Therefore, in addition to verbal evidence, non-verbal evidence (voice tones, expressions, body movements, etc.) was considered, such as facial expressions to get an idea of the level of joint pain and the veracity of the patient's assessment of the care team; tone of voice to determine the patient's level of confidence in being discharged from the hospital; and flexion/extension of the knee to determine the patient's current joint function. This really helped the researchers to better summarize the themes.

**Reviewer #1:** Table 1: report medians and not means. What does postoperative period mean? Report hospital length of stay data. If I guess LOS correctly, it seems quite high for a fast-track program. How was the number of chronic diseases determined?

**Author response:** Thank you for your valuable comments. First, we replaced the means with the medians and the postoperative period with the hospital length of stay(LOS) in the table 1. Secondly, our answer to the question that the LOS data seems quite high is as follows: in reviewing previous studies, the LOS for TKA under fast track has been reduced in the last decade in the USA^[1]^, Canada^[2]^, and the UK^[3]^, etc., to about 2-3 days postoperatively, but the average national LOS is still 3-5 days^[4, 5]^. The median LOS in this study was 3 days, which is an acceptable result. For the individual patient in this study who had LOS as long as 5 days, we can find that the patient was characterized by female, older than 80 years of age, and living with caregivers only, etc. For the patient, physicians consider moderately lengthening the LOS in order to prevent postoperative complications such as joint pain, inflammatory response, upright intolerance, and cognitive dysfunction, and to allow some of these high-risk patients to benefit from a planned extension of their hospital stays. Finally, regarding the determination of the number of chronic diseases, this study was obtained by reviewing patient case files and through verbal re-interviews. We pre-listed a number of common and highly prevalent chronic diseases, including hypertension, diabetes mellitus, arrhythmia, atherosclerosis, coronary heart disease, osteoporosis, eye disease, hearing impairment, stroke, obesity, cancer, gastritis, gout, bronchial asthma, and so on, and set up the option of "other" for patients to add.

References：

[1]	AUYONG D B, ALLEN C J, PAHANG J A, et al. Reduced Length of Hospitalization in Primary Total Knee Arthroplasty Patients Using an Updated Enhanced Recovery After Orthopedic Surgery (ERAS) Pathway[J]. J Arthroplasty, 2015,30(10): 1705-1709.

[2]	RAPHAEL M, JAEGER M, van VLYMEN J. Easily adoptable total joint arthroplasty program allows discharge home in two days[J]. Can J Anaesth, 2011,58(10): 902-910.

[3]	MAEMPEL J F, WALMSLEY P J. Enhanced recovery programmes can reduce length of stay after total knee replacement without sacrificing functional outcome at one year[J]. Ann R Coll Surg Engl, 2015,97(8): 563-567.

[4]	JENNY J Y, COURTIN C, BOISRENOULT P, et al. Fast-track procedures after primary total knee arthroplasty reduce hospital stay by unselected patients: a prospective national multi-centre study[J]. Int Orthop, 2021,45(1): 133-138.

[5]	STOWERS M D, MANUOPANGAI L, HILL A G, et al. Enhanced Recovery After Surgery in elective hip and knee arthroplasty reduces length of hospital stay[J]. ANZ J Surg, 2016,86(6): 475-479.

**Author change:** Table 1. Demographic and Characteristics of the Participants（N=21） (Page10-11)

| **Participants** | | **n=21** |
| --- | --- | --- |
| Sex | Male | 8 |
|  | Female | 13 |
| Age, years | Median | 68 |
|  | Range | 53-80 |
| Educational levels | Elementary school and below | 11 |
|  | Junior high school | 6 |
|  | High school (including junior college) | 2 |
|  | University (including college) and above | 2 |
| Marital status | Married | 17 |
|  | Widowed | 4 |
| Living arrangements | Only with spouse | 12 |
|  | With family members | 5 |
|  | Alone | 2 |
|  | With live-in care worker | 2 |
| Residence area | City | 12 |
|  | Rural | 9 |
| Hospital length of stay, days | Median | 3 |
|  | Range | 2-5 |
| Number of chronic diseases | 0 | 6 |
|  | 1~2 | 9 |
|  | 3 | 6 |

**Reviewer #1:** Line 204: submitting to medical authority seems a bit harsh. What about "preparing for discharge despite..."

**Author response:** Thank you for suggesting changes to the theme, after our consideration and communication, we unanimously agree with your suggestions and have now made the changes.

**Author change:** Preparing for discharge despite concerns about symptoms, (Page 12, Lines 206-207)

**Author change: Preparing for discharge despite concerns about symptoms** (Page 12, Line 210)

**Reviewer #1:** Line 256: replace gaming with managing.

**Author response:** Thank you for suggesting changes to the refinement of the theme of this study, which has now been revised.

**Author change:** Managing the rehabilitation difficulties, (Page 12, Lines 207-208)

**Author change: Managing the rehabilitation difficulties** (Page 14, Line 258)

**Reviewer #1:** Line 281: their

**Author response:** Thank you very much for pointing out the spelling errors in the words that appeared in our article, we apologize for this and have now made corrections and re-reviewed to ensure that there are no spelling problems with the words in this article.

**Author change:** the insufficient adaptation of their homes for post-surgery recovery frequently leads to delayed recuperation following hospital discharge. (Page 15, Line 286)

**Reviewer #1:** Line 386: do patients pay for hospital stay? Please add this context in the manuscript

**Author response:** Yes. Considering that the occupation of the bed, nursing services and treatment, patients are required to pay for the hospital stay. We will add the relevant context in the revised manuscript.

**Author change:** Second, besides the cost of restorative surgery, bed occupancy, nursing services, and medical treatment during postoperative hospitalization place a similar financial burden on the patient. Most cost-effectiveness studies(27, 28) have used data on length of stay to calculate costs. The length of a patient's postoperative hospital stay is considered a good indicator of healthcare costs, and the prospect of early discharge as a cost-effective rehabilitation option may motivate them to prepare for discharge. (Page 20, Lines 387-393)

27.	Jansen JA, Kruidenier J, Spek B, Snoeker B. A cost-effectiveness analysis after implementation of a fast-track protocol for total knee arthroplasty. KNEE. [Journal Article]. 2020 2020-03-01;27(2):451-8.

28.	Büttner M, Mayer AM, Büchler B, Betz U, Drees P, Susanne S. Economic analyses of fast-track total hip and knee arthroplasty: a systematic review. EUR J ORTHOP SURG TR. [Journal Article; Meta-Analysis; Systematic Review]. 2020 2020-01-01;30(1):67-74.

**Reviewer #1:** Line 409: The link with the results needs to be made clearer. Can you be more explicit on what subjects?

**Author response:** Thank you for your suggestion, we think it helps to explain the subjects of this study more clearly and we have rewritten this section based on the reviewers' comments. We offer this recommendation based on the findings of Theme 1(Preparing for discharge despite concerns about symptoms), which included the sub-themes of satisfaction with improved function and a sense of reborn joy, feelings of helplessness, stigmatization, and anxiety about prosthetic function. (Sub-themes included in theme are presented in the results of the revised manuscript.)

**Author change:** Secondly, it is essential to provide more comprehensive and detailed guidance to enhance patients' confidence in coping with feelings of helplessness, stigma, and anxiety about prosthetic function. (Page 21, Lines 411-413)

**Reviewer #1:** Line 419 and 422: please replace paranoid.

**Author response:** We appreciate your professional comments about our inaccurate terminology, and based on your suggestions, we have replaced it with “stubborn” in previous manuscript.

**Author change:** this strong intrinsic motivation may have been somewhat countered by patients’ stubborn attitudes towards pain management and the challenges posed by the external environment and social relationships. (Page 22, Lines 422-425)

**Author change:** some patients didn’t take pain management seriously and were stubborn about resisting using analgesics, (Page 22, Lines 425-426)

**Reviewer #1:** Line 421: probably not all patients.

**Author response:** Thank you for your question regarding the scope of the population involved. After further discussion, we realized that this was a misunderstanding, and for the sake of clarity, we will refer to "patients" as "some patients".

**Author change:** some patients didn’t take pain management seriously and were stubborn about resisting using analgesics, (Page 22, Lines 425-426)

**Reviewer #1:** Line 460: please replace infected.

**Author response:** Thank you for your suggestion about the poor use of words in this study, which we correct immediately, and we have decided to replace the word "infected" with the word "impacted".

**Author change:** This leads us to the idea that empathy and the ability to think differently in healthcare professionals can be learned and impacted as a teachable communication skill (Page 24, Lines 462-464)

**Reviewer #1:** Line 470: the results are limited to the Chinese health care system.

**Author response:** Thank you very much for revising the limitations of this study, we have changed the limitation of this study to the fact that the results are limited to the Chinese health care system.

**Author change:** Firstly, the participants were recruited exclusively from one hospital, and the results are limited to the Chinese health care system. (Page 24, Lines 472-474)

**Reviewer #2:**

**Reviewer #2:** Abstract: Very vaguely presented. Relevant details need to be added

**Author response:** We sincerely thank you for your valuable suggestions and have added details in each section of the abstract such as background, objectives, methods, results and conclusion respectively.

**Author change:**

Background

The fast-track based on evidence-based medicine, has dramatically reduced the length of stay for patients undergoing total knee arthroplasty (TKA). Therefore, patients must assume the responsibility for self-functional exercise and care as early as possible. Also, higher standards and expectations of care delivery have been set. Studies into patients’ experiences when faced with a discharge decision under a fast-track program are lacking.

Objectives

(1) Increase the knowledge about patients’ experiences of discharged from hospital via a fast-track process after TKA. (2) Explore what gaps exist in the current discharge preparation care service for TKA under fast-track and what can be improved.

Methods

A qualitative research design was chosen to conduct semi-structured face-to-face interviews with 21 patients from one Chinese hospital who successfully underwent TKA and received discharge orders. Interview data were meticulously analyzed, summarized and thematically distilled using Interpretative Phenomenological Analysis (IPA).

Results

Three themes emerged: a) Preparing for discharge despite concerns about symptoms-a sense of joy at discharge despite feelings of helplessness, stigmatisation, anxiety about prosthetic function. b) Managing the rehabilitation difficulties-vigilance is needed for the effects of medication use, environmental changes, and intimate relationships. c) Creating conditions for safe transition-compassionate bedside manner, listening to patients, and providing a humanized continuing care and referral services are important for safe transitions.

Conclusion

Findings suggest that patients undergoing fast-track TKA report good discharge preparation experiences. However, closer analysis reveals difficulties with this process and important directions in which discharge readiness care services can strive. (Page 2-3, Lines 23-52)

**Reviewer #2:** Intro: Very long. The purpose of the study is not clear. Relevant literature may be appropriately presented

**Author response:** Thank you for your valuable comments, we have re-read the Introduction and in the revised version, we have deleted repetitively expressed sentences to streamline the content. Also, we have made the purpose of this study more explicit, which contains two objectives.

Objective 1: To describe the discharge preparation experiences of patients after fast-track TKA and to clarify whether patients are consciously prepared for discharge. Objective 2: To explore what deficiencies exist in the current discharge preparation nursing service for TKA under fast-track and what can be improved. Moreover, we scrutinized the literature and added more references in the introduction section of the revised manuscript.

**Author change:** Using available 2000-2014 NIS figures, the U.S. projects that primary TKA is projected to grow 85%, to 1.26 million procedures, by 2030(3, 4).(Page 3-4, Lines 59-61)

**Author change:** Consequently, with the increasing number of TKAs in China, ~~and the corresponding strain on the healthcare system, there is a growing emphasis on ensuring patient well-being and optimizing healthcare services. Fast-track Surgery (FTS) has been shown not only to enhance surgical outcomes and increase patient satisfaction but also to reduce hospital costs. Consequently,~~ the concept ~~has gained extensive adoption in knee arthroplasty procedures~~ of fast track surgery (FTS) has been widely adopted in this field. (Page 4, Lines 68-69)

**Author change:** shorter hospital stays for TKA patients do not equate to reduced perioperative care but instead require significant organizational and administrative efforts by healthcare staff involved in patient discharge(12). (Page 4, Line 74)

12.	Hjort JD, Rud K, Kehlet H, Egerod I. Standardising fast-track surgical nursing care in Denmark. Br J Nurs. 2014 May 08;23(9):471-6. (Page 27, Lines 539-540)

**Author change:** ~~In actual clinical practice, discharge decisions are often primarily rely on objective criteria or prescribed lengths of stay(13, 14),with limited attention to patients' subjective experiences(15).~~(Page 4, Line 77)

**Author change:** ~~However, readiness for discharge is a multifaceted concept, which means that healthcare professionals may perceive a patient as more ready for discharge than the patients themselves do(16, 17).~~(Page 4, Line 77)

**Author change:** Previous studies consistently highlight that patients frequently don’t feel adequately prepared for discharge(13), with limited attention to patients' subjective experiences(14). Rushed discharges have been linked to elevated readmission rates(15), increased postoperative complications, and unexpected healthcare resource utilization(16). Weiss(15) and Coffey(17) have advocated that incorporating patients' own perspectives in evaluating discharge readiness reduces unplanned readmission and mortality rates, as well as optimizing the discharge planning process while improving patient satisfaction.(Page 4-5, Lines 77-84)

**Author change:** This study aims to describe patients' experiences of their discharge instructions following TKA under FTS in China to clarify whether patients consciously prepare for discharge. Additionally, this study attempts to explore what gaps exist in current discharge preparation care service and what could be improved. ~~The ultimate goal is to enhance health promotion efforts related to discharge preparation, thereby improving the overall patient experience and prognosis.~~ (Page 5, Lines 92-95)

**Reviewer #2:** Methods: "We purposively recruited patients": Does not read well. Pls modify the inclusion criteria

**Author response:** We found your suggestions very helpful and we have added more details on patient characteristics and modified the inclusion criteria. We have detailed that patients were first elective unilateral TKA due to advanced KOA, excluding fractures, rheumatoid arthritis, or revisions. In addition, all patients we included for interview were primary elective procedures.

**Author change:** The inclusion criteria were as follows: a) first unilateral TKA for advanced KOA; b) take primary elective surgery. c) receive fast-track program; d) receipt of a discharge order; e) demonstrate effective communication skills; and f) willingness to join the study and consent in writing. (Page 6-7, Lines 119-123)

**Reviewer #2:** Results: Does not follow a good scientific flow. Predominantly psychological issues have been discussed. No other clinical information of patients has been shared

**Author response:** Thanks for your suggestions, we have rewritten this section based on the reviewer's comments. During the process, we did our best to follow a good scientific flow to report only results-related content and to avoid excessive discussion in this section. Moreover, in order to present the results more clearly, sub-themes have been added to aid understanding (this section was shown further in S1 Table). Finally, in addition to emphasizing psychological issues, other details were included, which we have reflected in the revised manuscript.

**Author change: Satisfaction with improved functioning and a sense of reborn joy** (Page 12, Line 211)

**Author change:** In the interview reports, ~~we discovered that during the recovery from TKA, patients acknowledged a gradual regaining of physical independence despite experiencing pain and temporary post-surgical injuries.~~ patients expressed satisfaction with the improvement in knee function and a sense of reborn joy. Expressions such as “going home comes first” and “looking forward to being discharged” were frequently used. (Page 12, Lines 212-214)

**Author change:** ~~When considering the question of readiness for discharge, individuals~~ Patients tend to compare themselves to fellow patients or sense the beginnings of an impending discharge from conversations with nurses or rehabilitation physicians ~~within their immediate healthcare environment~~ when making their self-assessment. (Page 12, Lines 223-224)

**Author change:** ~~Participants seemed to sense the beginnings of an impending discharge from conversations with nurses or rehabilitation physicians and indicated that they were patiently waiting for final approval. Consequently, patients did not question the attending surgeon's discharge instructions.~~ (Page 13, Line 229)

**Author change: Feeling of helplessness** (Page 13, Line 233)

**Author change:** ~~Throughout the interviews, alongside their expressed eagerness for an early discharge, participants also conveyed internal concerns. For example,~~ The influx of ~~overwhelming~~ information or heightened sensitivity to physical symptoms generated a sense of vigilance and uncertainty among participants who were overwhelmed. (Page 13, Lines 234-236)

**Author change: Stigmatisation** (Page 13, Line 245)

**Author change: Anxiety about prosthetic function** (Page 14, Line 251)

**Author change: Highly motivated and currently on the road to recovery** (Page 14, Line 259)

**Author change: Medicines management** (Page 15, Line 272)

**Author change: Environmental changes** (Page 15, Line 284)

**Author change: Strained social and intimate relationships** (Page 16, Line 304)

**Author change:** ~~Family members or close friends continue to play a crucial role in providing comfort, boosting patient confidence, and facilitating engagement in activities during the recovery process. However,~~ The majority of participants reported a significant reduction in ~~their social interactions~~ socializing with friends and family relatives due to limitations in mobility and feelings of frustration. (Page 16, Lines 305-306)

**Author change:** ~~Participants’ spouses often take on the important role of providing informal care free of charge.~~ An “ideal spousal model of recovery” ~~appears to be their preferred approach. In order to provide better care,~~ resulted in spouses often taking on the important role of providing informal care free of charge, and participants mentioned that they suspended some physical intimacy to recover from the illness, ~~and although these sacrifices favoured the recovery of the participants from their illnesses, they~~ but that did have an impact on the intimacy of the couple. (Page 17, Lines 311-314)

**Author change: The caring bedside manner of the nursing staff** (Page 17, Line 328)

**Author change: Listening to patients** (Page 18, Line 342)

**Author change:** ~~Attending to patients' voices is a pivotal aspect of healthcare professionals adopting a different mindset, and~~ In this study, participants emphasized the significance of the care team listening to their input and desires, as evidenced by communicating the timing of medication changes and choosing more appropriate means of communicating health information. (Page 18, Lines 343-345)

**Author change: A humanised continuing care and referral services** (Page 19, Line 357)

**Author change:** ~~Patient discharges can make nurses' jobs hectic and logistically complicated, throwing the discharge sequence into chaos.~~ (Page 19, Line 358)

**Reviewer #2:** Discussion: Very superficial. The manuscript does not have a scientific flow. The purpose of the study is not clarified. Inadequate details have been furnished

**Author response:** We sincerely thank you for your careful reading, and we have fully considered your suggestions and made our best efforts to revise the manuscript, rewrite it according to the scientific process, clarify the purpose of this study and provide relevant details.

**Author change:** This study presents the discharge readiness experience of patients undergoing fast-track TKA. It adds valuable knowledge about the understanding of how patients treated with TKA under fast track setting when facing discharge, in addition to revealing the difficulties patients face, it also adds some positive responses about clinical care practices. (Page 20, Lines 373-377)

**Author change:** To clarify whether patients were consciously ready to be discharged, ~~It was apparent that~~ the majority of participants reported favorable postoperative recovery experiences and expressed a readiness to be discharged from the hospital. (Page 20, Lines 378-380)

**Author change:** Second, besides the cost of restorative surgery, bed occupancy, nursing services, and medical treatment during postoperative hospitalization place a similar financial burden on the patient. Most cost-effectiveness studies(27, 28) have used data on length of stay to calculate costs. The length of a patient's postoperative hospital stay is considered a good indicator of healthcare costs, and the prospect of early discharge as a cost-effective rehabilitation option may motivate them to prepare for discharge. (Page 20, Lines 387-393)

**Author change:** Significantly, ~~while patients shared typical stories of positive feedback regarding their impending discharge, a notable point of concern emerged when they were reminded that, upon leaving the hospital, they would be responsible for managing their own medication, engaging in functional exercises, and self-care without the immediate guidance of healthcare professionals.~~ behind their positive response to the discharge, it became evident that various psychological issues were at play, including feeling of helplessness due to uncertainty about the medical condition, fear of social stigma, and heightened concerns about knee prosthesis. (Page 20-21, Lines 393-396)

**Author change:** The same paradox was found in the study by Woolhead(29),where individuals reported their outcome from TKR as good, but further discussion revealed concern and discomfort with continuing pain and mobility difficulties. (Page 21, Lines 396-399)

**Author change:** Such findings highlight a number of important issues related to the evaluation of discharge readiness. ~~What’s more,~~ A mixed study(30) conducted in Malaysian ~~context, in~~ exploring the needs of orthopedic patients at discharge, found that there were emotional, physical and spiritual preparation needs. What’s more,in a study of nurses' and patients' perceptions of the discharge readiness, respectively, it was found that nurses rated discharge readiness higher than patients did(31). (Page 21, Lines 399-404)

**Author change:** Secondly, it is essential to provide more comprehensive and detailed guidance to enhance patients' confidence in coping with feelings of helplessness, stigma, and anxiety about prosthetic function. (Page 21, Lines 411-413)

**Reviewer #2:** Conclusion: Long and not very clear.

**Author response:** We sincerely thank you for pointing out our shortcomings, and we have revisited the relevant elements of our conclusions and have streamlined and clarified the conclusions of this study in the revised manuscript.

**Author change:** Findings suggest that patients undergoing fast-track TKA report good discharge preparation experiences. However, closer analysis reveals difficulties with this process, highlighted by psychological activities such as feelings of helplessness, stigma, and anxiety associated with uncomfortable symptoms. Pain control, attention to environmental modifications and strengthening of social and family support are important to improve postoperative management. Finally, discharge preparation nursing services can continue to work on a cordial bedside manner, listening to the patient, and humanizing the continuum of care or referral services. (Page 25-26, Lines 499-506)
