# Supplementary material for: Are patients ready for discharge from the hospital after fast-track total knee arthroplasty?-A qualitative study
Source: PLoS One. 2024 May 29;19(5):e0303935. doi: 10.1371/journal.pone.0303935 (PMC11135671; doi:10.1371/journal.pone.0303935)
Supplement: Attachment — Submitted filename: Response to Reviewers.docm [file pone.0303935.s007.docm]

Robert Jeenchen Chen, PhD

Academic Editor

PLOS ONE

Re:	PONE-D-23-33287

Are patients ready for discharge from the hospital after fast-track total knee arthroplasty? -A qualitative study

**Additional editor comments**

Thank you for submitting your manuscript to PLOS ONE. After careful consideration, we feel that it has merit but does not fully meet PLOS ONE’s publication criteria as it currently stands. Therefore, we invite you to submit a revised version of the manuscript that addresses the points raised during the review process.

**Author response:** Thank you for the opportunity to submit a revised version of the manuscript. We greatly appreciate the reviewers’ comments and consider addressing these have led to an improved manuscript. We can confirm the manuscript meets PLOS ONE's style requirements, including those for file naming. Please find our response to the reviewers’ comments below. We have also included the manuscript changes and used red type below to highlight the relevant changes to the manuscript text.

**Reviewer #1**

**Reviewer #1:** Line 272: replace medicines with medication

**Author response:** Thanks for your kind suggestions, which are valuable for improving the accuracy of the manuscript. We have made a revision to this.

**Author change:** Medication ~~Medicines~~ management (Page 15, Line 281)

**Reviewer #1:** Line 480 and 483: I don't think stubborn is the right word either in the context of patient-centered care. The patient's feelings should be more respectfully acknowledged, and a term more patient-centered should be used. I would suggest rephrasing to simply use the patient's resistance to use analgesics, and remove any paternalistic terms.

**Author response:** Thank you very much for your suggestions, and in the revised manuscript we have paid further attention to the use of word and removed some of the paternalistic terms

**Author change:** However, this strong intrinsic motivation may have been somewhat countered by patients’ resistance to use analgesics ~~stubborn~~ ~~attitudes towards pain management~~ and the challenges posed by the external environment and social relationships. (Page 22, Lines 423-424)

For example, some patients didn’t take pain management seriously and ~~were stubborn about resisting~~ resisted using analgesics, despite repeated messages that pain should be nipped in the bud. (Page 22, Lines 426)

**Reviewer #2:** We appreciate the authors hard work. The authors goal is to evaluate whether the patients are discharge ready for fast-track discharges. However, the manuscript does not really serve the goal. The manuscript is less scientific; and more anecdotal in its approach

**Author response:** Thank you very much for reading this paper carefully and for making meaningful recommendations. In response to the goal of this study, the primary aim of this study was 1) to increase the knowledge about patients’ experiences of discharged from hospital via a fast-track process after TKA. The secondary aim was 2) to explore what gaps exist in the current discharge preparation care service for TKA under fast-track and what can be improved.

In terms of evaluating whether the patients are ready for fast-track discharges, this study was a qualitative study in nature and could not really achieve this goal, which is related to the methodology that distinguishes qualitative studies from quantitative studies. This study was only able to discern that patients expressed a good experience of discharge readiness, and relevant inappropriate statements in the text were modified or removed in this manuscript.

Regarding the less of scientific in this manuscript, and more anecdotal in its approach. After receiving comments from the reviewer, the joint research team revisited and understood the thick descriptive content and revised the presentation of the data excerpts provided in the results.

**Author change:** This study aims to describe patients' experiences of ~~their discharge instructions~~ discharged from hospital following TKA under FTS in China ~~to clarify whether patients consciously prepare for discharge~~.(Page 5, Lines 92-93)

The interview process naturally provided a comprehensive understanding of

~~whether participants were prepared~~ patients' experiences of preparing for discharge,(Page 12, Line 206)

~~To clarify whether patients were consciously ready to be discharged, t~~The majority of participants reported favorable postoperative recovery experiences and expressed a readiness to be discharged from the hospital.(Page 20, Line 378)

**Author change:**

In the interview reports, patients expressed satisfaction with the improvement in knee function ~~and a sense of reborn joy~~.(Page 12, Line 213)

*“I feel like I'm healing day by day...(The third day after the surgery)” (ZC, Male, 68 years old, Post-op day 4)*

*“my surgeon told me that my test results were good, and rehab exercises were on track...” (GWX, Male, 57 years old, Post-op day 3)*(Page 12, Lines 214-217)

*“The guy next to me had his surgery a day before me, and he got sent home yesterday...~~I’m a bit worried and scared... But~~ I guess it’s probably my turn to go home.” (ZYJ, Female, 63 years, live only with spouse, Post-op day 3)*(Page 12, Lines 221-223)

*“The physician said that I can bend (90°) and straighten (180°) my knee now, I would be out of the hospital soon.” (LW, Male, 69 years old, Post-op day 4)*(Page 12, Lines 224-225)

As the topic of discharge came up, they felt a sense of reborn joy.(Page 12, Line 226)

*“The surgery went well... ~~I feel way stronger than before (used to struggle with stairs), although my knee hurts a bit now, it’s manageable, and I'm recovering nicely...~~ Getting home is what I’m really looking forward to.” (ZTA, Male, 66 years, live only with spouse, Post-op day 3)* (Page 13, Line 229)

*“~~Prep started right after the surgery... Those walks kept getting longer, and I got tired, but the docs and nurses were really on top of things...~~ I’m all set to head home now, going home comes first.” (ZYX, Female, 54 years, live only with spouse, Post-op day 2)*(Page 13, Line 231)

**Author change:**

*“It happened so quickly~~, I didn’t even realize I was suddenly kicked out of the hospital, and now I feel like I’m just winging it~~... If only they had let me stay one more day, I think I could have absorbed all this info...” (SXQ, Female, 69 years, live alone, Post-op day 3)*(Page 13, Line 237)

*“~~Now it’s all up to me... There are still plenty of things~~ I’m uncertain about... I ~~stand for a while doesn’t feel quite right,~~ cause the skin on this leg is numb and doesn’t quite feel like mine, but maybe that’s just part of the deal.” (WTY, Female, 61 years, live with family members, Post-op day 2)* (Page 13, Line 240)

Participants also highlighted their feelings of helplessness in the face of future self-care.

*“I'm not so sure I would have done subsequent rehab movements on my own. A sense of being left to its own devices.” (DJ, Female ,71 years old, Post-op day 3)*(Page 13, Lines 243-246)

**Author change:**

*“~~I'm not sure if folks will treat me differently when I return because of this artificial knee.~~ They might think I’m in the same boat as someone with a disability.” (ZYX, Female, 54 years, live only with spouse, Post-op day 2)*

(Page 14, Line 250)

In turn, they argued that the majority of people on the ward had undergone joint surgery and that there were similarities between them, therefore they would not be viewed differently.

*“In the hospital, we're all in the same boat, and there's no judgment like that（discriminated against）”. (CBC, Female, 78 years old, Post-op day 3)*(Page 14, Lines 252-256)

**Author change:**

*“I'm kinda scared that if I push it too hard, I might end up needing another replacement...” (ZYJ, Female, 63 years old, Post-op day 3)*(Page 14, Lines 264-265)

*“This surgery is a big deal in my life, I’m absolutely committed to my post-surgery rehab... ~~I’m confident I can keep up with the rehab routine I’ve been taught.~~” (GXP, Female, 68 years, live only with spouse, Post-op day 3)*

*“Post-op rehab is a big deal, and I can't afford to lose in this battle.”(YM, Male, 74 years old, Post-op day 3)*(Page 15, Lines 278-280)

**Author change:**

These include a biased comprehension of medication dosages, such as excessive caution or relying on painkillers.(Page 15, Lines 283-284)

*“I barely noticed the minor pains, and as long as it’s not particularly painful, I’ll opt out of pain medication because painkillers are a chemical. ~~I had to hobble around the room with a walker, and I’ll admit, I was feeling pretty scared.~~” (ZWQ, Female,73 years, live alone, Post-op day 4)*(Page 15, Lines 285-287)

*“I'll admit, I was feeling scared. Requested my doctor to give me an extra dose of painkillers...”(LYQ, Male, 56 years old, Post-op day 2)*(Page 15, Lines 288-289)

~~and the fact that most~~ Actually, most patients people do not realise that the pain can be intense, especially at night. (Page 15, Line 290)

*“Well rested is important, (The first night after surgery) I didn’t really get into a deep sleep and the pain got worse, it’s quite a struggle. ~~Sometimes, pushing it can be more of a burden.~~” (XZZ, Female, 68 years, live with other family members, Post-op day 3)*(Page 16, Lines 292-294)

*“I mean... at home there isn’t a strict no-smoking policy like there is in hospitals, where everything around you has handles and is sterilised regularly. ~~At home, there are no handles in the bathroom and I’m always worried about accidental injuries or infected wounds when I’m walking around.~~” (DJ, Female, 71 years, live only with spouse, Post-op day 3)*(Page 16, Lines 300-302)

*“That walker won’t fit in our bathroom at home, and I’m worried I might take a tumble without it. ~~It’d be great to have some kind of handrail I can grab onto when I’m moving around and getting up from the toilet.~~” (JH, Female, 53 years, live only with spouse, Post-op day 2)*(Page 16, Lines 303-304)

*“My doc and nurse told me to stick to a high-protein and light diet~~, and they kept a close eye on what I was eating in the hospital.~~ But you know, I’m a fan of spicy food. ~~That’s just how we roll when it comes to eating habits.~~” (XLE, Male, 62 years, live only with spouse, Post-op day 3)*(Page 16, Lines 307-308)

*“~~I really appreciated the calls and messages of support from my friends when they heard about my major surgery this time, but~~ I won’t be attending for too many gatherings because I hardly go out on my own anymore.” (YS, Male, 67 years, live only with spouse, Post-op day 3)*(Page 16, Lines 312-313)

*“My son burned through all his saved-up vacation days~~. Actually, he didn’t really know how to help me get up or out of bed. He even made me a cup of tea, which I can’t drink right now~~... ~~But turning him down would feel impolite, especially since~~ he’s given up his precious time and spent a lot of money.” (LW, Male, 69 years, live with other family members, Post-op day 4)*(Page 17, Lines 325-327)

*“my daughter was busy with work...but she put a lot of effort into this.” (LYQ, Male, 56 years old, Post-op day 2)*(Page 17, Lines 328-329)

**Author change:**

*“They actually came right to my bedside, kept repeating instructions in case I forgot, treating me like one of their own family... ~~They were with me from the surgery all the way to when I was ready to leave.~~” (GYH, Female, 80 years, live with live-in care worker, Post-op day 5)*(Page 18, Lines 338-340)

*“~~Later that morning, they said they’d be back in an hour or two to switch up my meds, and the rehab person was scheduled to come by at 10:00 a.m.~~ I didn’t want to start rehab before the med change because I knew it would make the pain worse... The nurse understood my concern and shifted the schedule.” (XGB, Female, 69 years, live only with spouse, Post-op day 3)*(Page 18, Lines 348-350)

*“The nurses handed us a bunch of booklets, but ~~I had a hard time with them because~~ I’ve got presbyopia. So, ~~when I asked the nurses about it,~~ they hooked me up with a bunch of rehab videos... ~~I gave it very good in my nurse’s review.~~” (ZTA, Male, 66 years, live only with spouse, Post-op day 3)*(Page 18, Lines 351-353)

Participants reported that when the care team learnt about their needs, they were given special consideration and felt valued.(Page 18, Lines 354-355)

*“The benches in the ward were too low for me, probably because of my height (187 cm)...I mentioned this to the nurse, and she somehow managed to find me a taller chair.” (XLE, Male, 62 years old, Post-op day 3)*(Pages 18-19, Lines 356-358)

*“~~I’ll be getting out of here soon, and I know the nurses are swamped with their to-do list.~~ I was wondering if I could have their contact info because they know my health situation better than anyone else.” (SXQ, Female, 69 years, live alone, Post-op day 3)*(Page 19, Lines 363-364)
